# Paying Patience Back to *Impatiens* (Balsaminaceae): Hidden Biodiversity in the Qinling‐Bashan Mountains, China

**DOI:** 10.1002/ece3.72081

**Published:** 2025-09-15

**Authors:** Zhang‐Jie Huang, Pei‐Liang Liu

**Affiliations:** ^1^ Key Laboratory of Resource Biology and Biotechnology in Western China, Ministry of Education Northwest University Xi'an China; ^2^ Bazi Collection & Botanical Garden Mengzi Yunnan China

**Keywords:** biodiversity, clarification, species‐rich genus, specimen, taxonomy

## Abstract

The Qinling‐Bashan Mountains mark the natural division of the temperate and subtropical climate zones in China. Though they are known as a biodiversity hotspot in China, the species diversity in these areas has been underestimated, especially in the species‐rich genus *Impatiens* whose northmost distribution range meets here. The taxonomy of *Impatiens* is fraught with difficulties because of the complex floral structures. In this study, integrated approaches including comprehensive literature consultation, specimen inspection, careful morphological comparison, and phylogenetic analysis reveal hidden biodiversity of *Impatiens* in the Qinling‐Bashan Mountains. Five new species are described here, and five species are reported from the Qinling‐Bashan Mountains for the first time. Additional notes are also provided to elucidate taxonomic ambiguities.

## Graphical abstract

This article reveals the hidden biodiversity of *Impatiens* from the Qinling‐Bashan Mountains, China. Five new species are described here, and five species are reported from the Qinling‐Bashan Mountains for the first time.

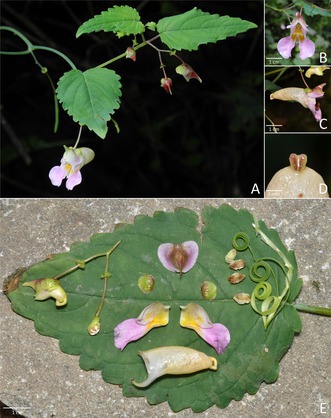

## 1 Introduction

The Qinling‐Bashan Mountains extend about 800 km from west to east in western‐central China, functioning as a transitional region between temperate and subtropical zones and a natural boundary of the North and South China. Such geographic traits, along with microhabitat differentiation, give rise to high biodiversity receiving great attention and interest in biogeographic research. The number of seed plants documented in Qinling is 3839 (Li and Li 2013) and that in Bashan is 3100 (Jia et al. 2014). Moreover, new species and new records have been continuously reported from these tremendous mountains (e.g., Fu et al. 2020; Jiang et al. 2022; Xun et al. 2022; Li et al. 2024; Wang et al. 2024), indicating that the biodiversity of these regions is still underrated.

*Impatiens* L. (Balsaminaceae) is a large genus comprising over 1100 species worldwide (POWO 2025), particularly centered in tropical Africa, Madagascar, southern India, Sri Lanka, the eastern Himalaya, and Southeast Asia (Yuan et al. 2004), and more than 350 among them are found in China (Yuan et al. 2022). Li and Li (2013) recorded eight *Impatiens* species, and Jia et al. (2014) reported 13 species from Qinling and Bashan, respectively. On the basis of our multiple fieldwork since the year 2008, the treasure of herbarium specimens and the advanced phylogenetic reconstructions (Fujihashi et al. 2002; Yuan et al. 2004; Janssens et al. 2006; Janssens, Wilson, et al. 2012; Yu et al. 2016), this paper describes five new species of *Impatiens* and reports five species from the Qinling‐Bashan Mountains for the first time.

## 2 Materials and Methods

### 2.1 Morphological Comparison

Physical specimens and high‐resolution images of concerned *Impatiens* species were examined in herbaria B, E, G, HEAC, IBSC, K, KUN, LE, NY, P, PE, TIE, US, W, WU, WNU, WUK, and XBGH (herbarium code follows Thiers 2024). Careful observations of living plants were conducted in the wild in the Qinling–Bashan Mountains and adjacent provincial regions including Chongqing, Gansu, Henan, Hubei, Ningxia, Shanxi, and Sichuan from 2008 onward. Morphological terminology follows Song et al. (2005), Chen et al. (2007) and Beentje (2016). Specimens and materials from living plants were documented, examined, and measured using a Nikon D7500 digital camera equipped with a Nikon AF‐S VR Micro‐NIKKOR 105 mm f/2.8G ED lens and ImageJ v1.54g (National Institutes of Health, USA). Floral traits such as the number of flowers per inflorescence, the shape and position of bracts, as well as the size, shape, and color of lateral sepals, lower sepal, dorsal petal, and lateral united petals are essential for the identification and species delimitation of *Impatiens* (Janssens, Smets, and Vrijdaghs 2012; Ruchisansakun et al. 2016; Yu et al. 2016). Therefore, we conducted detailed measurements of these characteristics.

### 2.2 Phylogenetic Reconstruction

The nuclear ribosomal ITS (Yuan et al. 2004) and the plastid *atpB*‐*rbcL*, *trnL*‐*F*, and *psbA*‐*trnH* (Janssens et al. 2006; Shajitha et al. 2016) sequences were used to reconstruct the phylogenetic relationship. New sequences of 70 samples representing 35 species were generated in this paper. DNA sequence data of 84 (nuclear) and 114 (plastid) species were downloaded from GenBank (see Table A1 for GenBank numbers). A total of 105 (nuclear) and 128 (plastid) *Impatiens* species were included in the current phylogenetic analyses, covering all subgenera and sections in Yu et al. (2016). *Hydrocera triflora* was selected as the outgroup.

Genomic DNA was extracted from dried leaf material using Trelief Hi‐Pure Plant Genomic DNA Kit (TSINGKE Biological Technology, Ezhou) or DNeasy Plant Mini Kit (QIAGEN, Hilden). PCRs were conducted using the primers (White et al. 1990; Taberlet et al. 1991; Sang et al. 1997; Janssens et al. 2006). PCR products were sequenced in both directions using the same primers.

Sequences were assembled in Geneious v9.0.2 (Kearse et al. 2012). Alignments were achieved using MUSCLE v3.8.425 (Edgar 2004) as implemented in Geneious, followed by minor manual adjustments. Gaps were treated as missing data. Sequences of the three plastid sequences were concatenated using PhyloSuite v.1.2.3 (Zhang et al. 2020). The best‐fit nucleotide substitution models and the partition scheme for the concatenated plastid data were selected via IQ‐TREE v1.6.7 (Nguyen et al. 2015), and were used in the Maximum likelihood (ML) analyses and the Bayesian inference (BI). The model for nrITS was GTR + F + I + G4. For the plastid data, IQ‐TREE found a two‐partition scheme: *atpB*‐*rbcL* plus *trnL*‐*F* for partition 1, while *psbA*‐*trnH* alone for partition 2, and the models for the two partitions were GTR + F + G4 and HKY + F + G4, respectively. ML analyses were performed via IQ‐TREE for 10,000 ultrafast bootstraps. BI analyses were conducted using MrBayes v3.2.6 (Ronquist et al. 2012) with 10 million generations. Trees and parameters were sampled every 1000 generations, and the initial 25% sampled data were discarded as burn‐in. Any clade with a posterior probability (PP) smaller than 0.5 was shown as a polytomy. The PPs and the bootstrap support percentages from the ML analyses were labeled on the trees. Phylogenetic trees were visualized in iTOL v.7 (https://itol.embl.de).

## 3 Results

### 3.1 Morphology

The new species *I. perforata* is similar to *I*. *conchibracteata, I*. *compta*, and *I*. *uncata* by having a saccate lower sepal and an incurved short spur. However, *I. perforata* differs from the latter by its cupuliform, amplexicaul bracts; the abaxial midvein of the dorsal petal has a long and broad rostellum in the middle.

*Impatiens notolopha* (Figure A3) is diagnosed by relatively large floral size (lateral sepals 3.5 mm; lower sepal 5.5 mm wide, 2.5 mm long, spur 12–13 mm; dorsal petal 4.5 mm, lateral united petals 8 mm long) (Maximowicz 1889). The new species *I*. *aciformis* has considerably smaller flowers (lateral sepals ca. 3 mm; lower sepal including spur ca. 6 mm; dorsal petal ca. 2 mm; lateral united petals ca. 3.5 mm). We also notice that *I*. *aciformis* and *I*. *notolopha* are largely allopatric. They overlap in Jiuzhaigou Valley, Sichuan (both were sampled on the trees), but no intermediate form is observed in the populations, implying the potential reproductive isolation between them. The different sizes of flowers may interact with varied pollinators, which in turn triggers speciation.

Morphologically, the new species *I. zhui* is similar to *I*. *pterosepala* and *I. dasyvexilla* (Figure A7), but can be differentiated from them by its dorsal petal with a long rostellum and the whole plant glabrous.

The new species *I. kuntsunii* is rather common in the Qinling Mountains and adjacent areas in Ningxia and Sichuan. It resembles *I. noli‐tangere* (Figure A5) but can be readily distinguished by a set of floral characters (see the taxonomic treatment).

*Impatiens latebracteata* has orbicular bracts with a dentate margin, and asymmetric lateral sepals (Hooker 1910). In contrast, *“I*. *latebracteata”* from the Qinling Mountains has ovate to broadly ovate bracts with an entire margin, and even lateral sepals. We name the Qinling plants *I*. *cordibracteata*. It is noteworthy that these two species are completely allopatric; *I*. *latebracteata* is found in Sichuan Province in the south, whereas *I*. *cordibracteata* is found in Shaanxi Province in the north.

### 3.2 Molecular Phylogenetic Analysis

**FIGURE A1:**
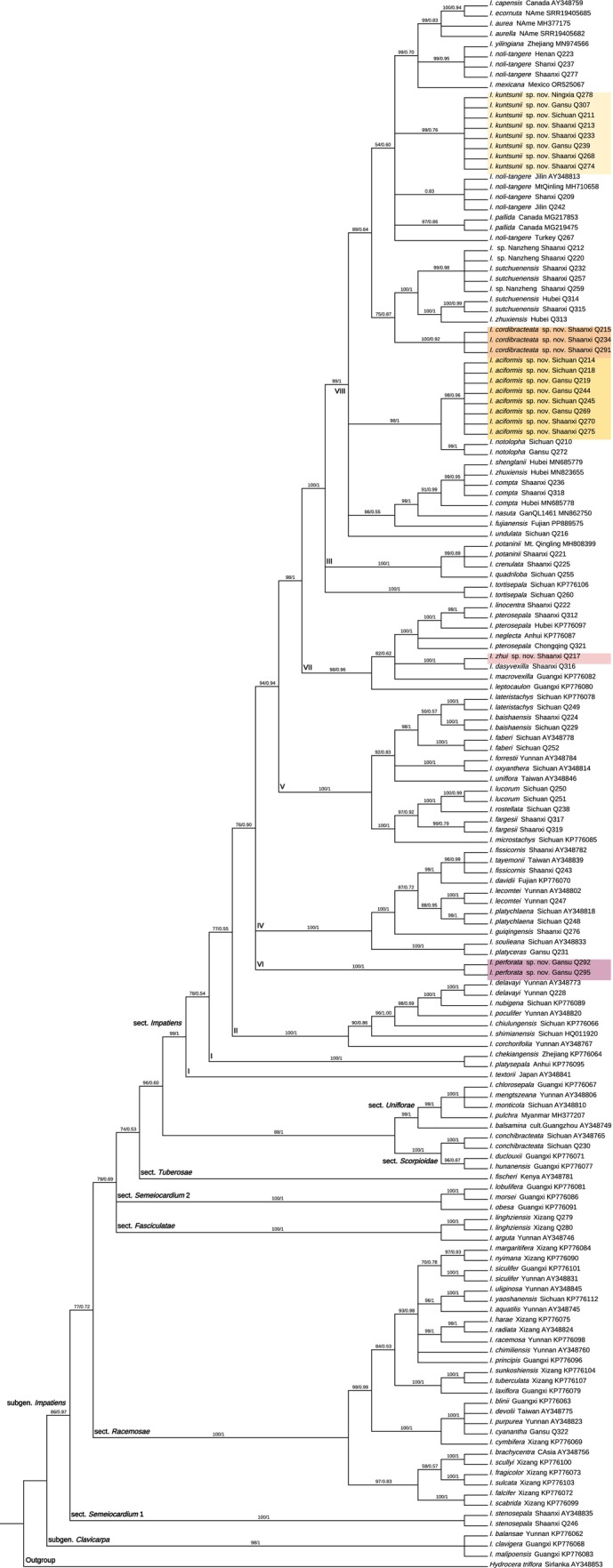
Bayesian phylogenetic tree inferred from nrITS sequence data. Values above branches are maximum likelihood bootstrap percentages/Bayesian posterior probabilities. New species are shown with colorful backgrounds.

**FIGURE A2:**
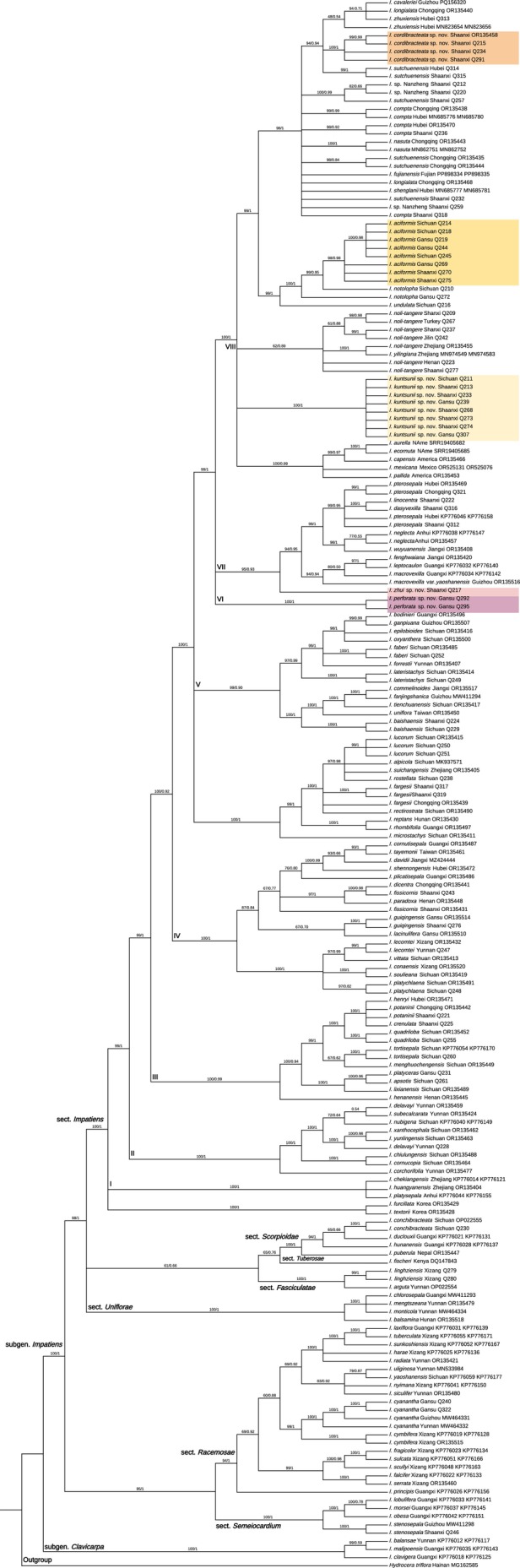
Bayesian phylogenetic tree inferred from concatenated data of three plastid sequence segments (*atpB‐rbcL*, *trnL‐F*, and *psbA‐trnH*). Values above branches are maximum likelihood bootstrap percentages/Bayesian posterior probabilities. New species are shown with colorful backgrounds.

**FIGURE A3:**
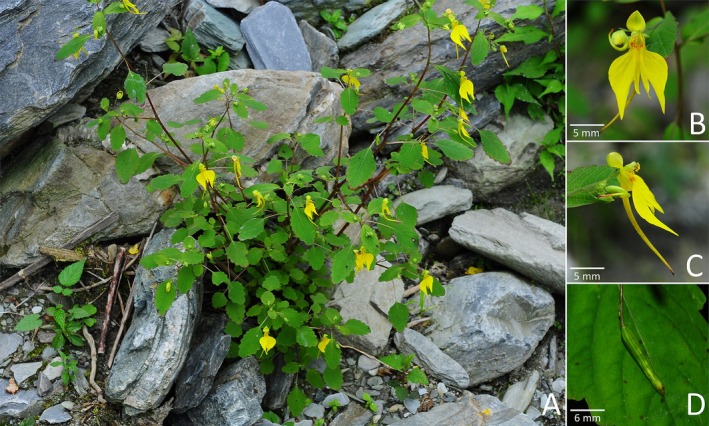
*Impatiens notolopha*: (A) habit, (B) front view of the corolla, (C) side view of the corolla, (D) fruit.

A total of 158 (nuclear) and 194 (plastid) samples were used in this study. The final aligned positions of the nuclear ITS dataset were 800 base pairs (bps), and the concatenated plastid *atpB*‐*rbcL*, *trnL*‐*F*, and *psbA*‐*trnH* dataset was 2527 base pairs (bps). We did not concatenate the nuclear and plastid datasets because the nuclear and plastid trees show some topological incongruences. In both the nuclear and plastid trees, *Impatiens* is divided into two subgenera, namely subgenus *Impatiens* and subgenus *Clavicarpa*. *I*. subgen. *Impatiens* can be divided into seven sections recovered by Yu et al. (2016) (Figures A1, A2).

**FIGURE 1:**
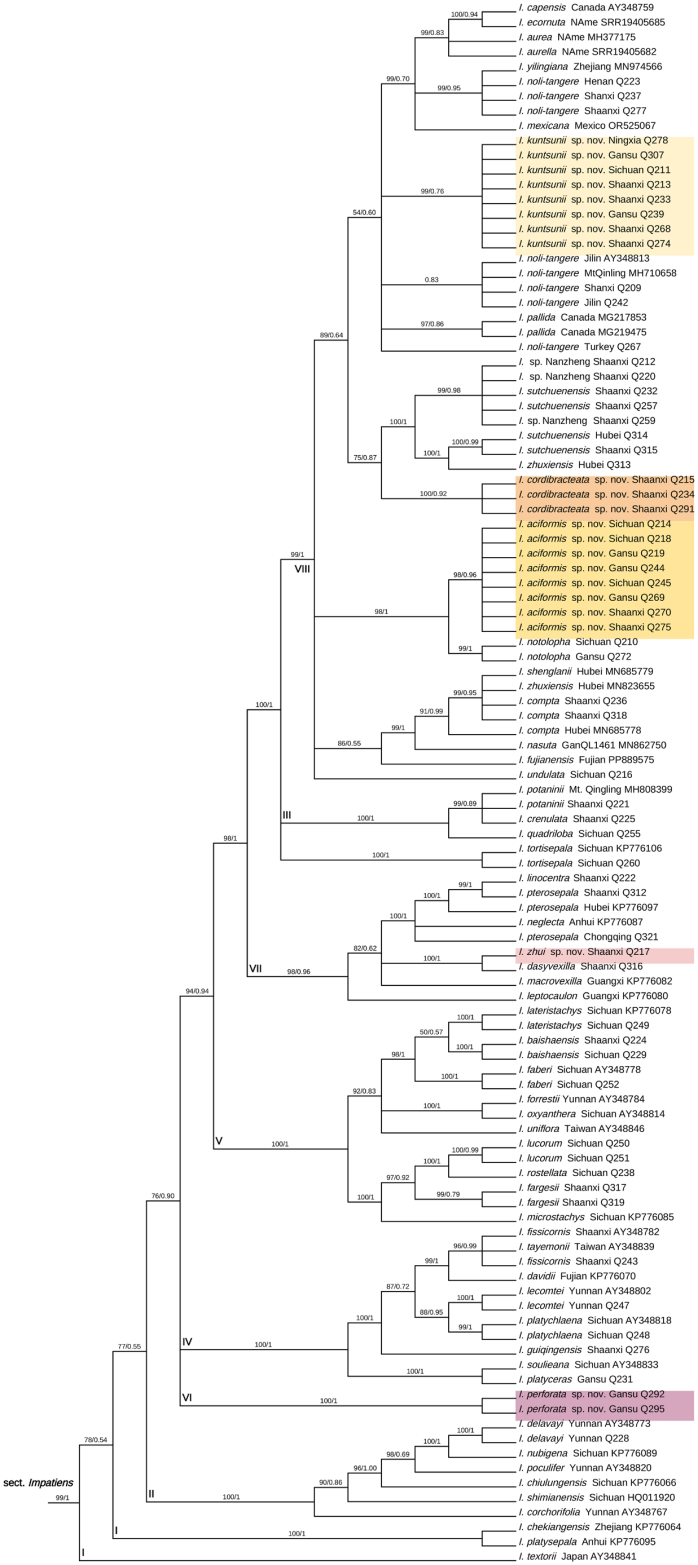
Bayesian phylogenetic tree of *Impatiens* sect. *Impatiens* inferred from nrITS sequence data. Values above branches are maximum likelihood bootstrap percentages/Bayesian posterior probabilities. New species are shown with colorful backgrounds.

**FIGURE 2:**
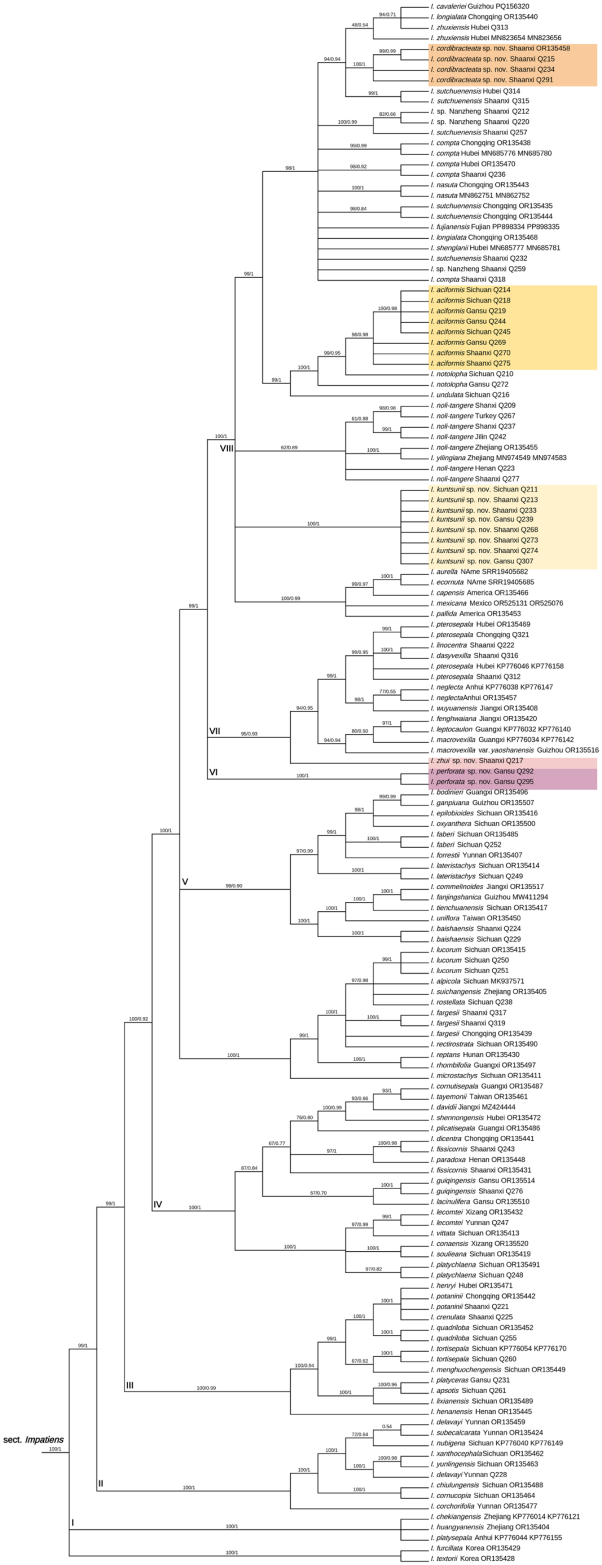
Bayesian phylogenetic tree of *Impatiens* sect. *Impatiens* inferred from concatenated data of three plastid sequence segments (*atpB*‐*rbcL*, *trnL*‐*F* and *psbA*‐*trnH*). Values above branches are maximum likelihood bootstrap percentages/Bayesian posterior probabilities. New species are shown with colorful backgrounds.

All newly described species belong to section *Impatiens*. Section *Impatiens* is made up of eight clades (Figures 1, 2). The new species *I. perforata* forms a single lineage (Clade VI) with strong support (ML/BI = 100/1, Figures 1, 2) on both trees. Samples of the new species *I*. *aciformis* from multiple localities form a strongly supported lineage within Clade VIII (ML/BI = 98/0.96 on the nuclear tree Figure 1, ML/BI = 98/0.98 on the plastid tree Figure 2). *I*. *aciformis* is sister to *I*. *notolopha* on the nuclear tree (ML/BI = 98/1, Figure 1). On the plastid tree, *I*. *notolopha* is paraphyletic. The new species *I. zhui* is sister to *I*. *dasyvexilla* on the nuclear tree (ML/BI = 100/1, Figure 1) in clade VII. On the plastid tree, however, *I. zhui* is at the base of clade VII (ML/BI = 95/0.93, Figure 2). On both trees (Figures 1, 2), samples of *I. kuntsunii* from different localities form a single lineage (nuclear, ML/BI = 99/0.76, Figure 1; plastid, ML/BI = 100/1, Figure 2) in Clade VIII. Samples of *I*. *cordibracteata* from different populations form a well‐supported lineage on the trees (nuclear, ML/BI = 100/0.92, Figure 1; plastid, ML/BI = 100/1, Figure 2).

## 4 Discussion

Our detailed field observations and phylogenetic analyses support the recognition of *I. aciformis*. However, previous researchers misidentified this new species as *I*. *notolopha*. *I. aciformis* is widely distributed in Shaanxi, Gansu, Sichuan, Ningxia, and Qinghai in China. This may be a reason for misidentification. The plate of “*I*. *notolopha*” in *Flora Tsinlingensis* (Ho 1981) shows a considerably small flower (lateral sepals ca. 3 mm; lower sepal including spur ca. 6 mm; dorsal petal ca. 2 mm; lateral united petals ca. 3.5 mm), which is actually *I. aciformis*.

The new species *I. kuntsunii* reported in this paper has been collected many times long before being recognized aright. The specimens deposited at PE and WUK are either misidentified as *I. noli‐tangere* or left undetermined. This might be because the pressing and drying processes make the floral parts of *Impatiens* brittle, which hinders accurate reconstruction and identification of the specimens. Experience of careful field observation of fresh flowers as well as systematic phylogenetic analyses will certainly be of great help to the taxonomy of *Impatiens*.

We also report five species of *Impatiens* from the Qinling‐Bashan Mountains for the first time (see Section 6). Together with the five new species, these new findings reveal part of the hidden biodiversity in the Qinling‐Bashan Mountains. The new *Impatiens* species described in this article are included in the identification key provided in the Appendix, accompanied by a distribution map of these species (Figure A11).

## 5 Taxonomic Treatments

### 5.1 *Impatiens perforata* Z. J. Huang & P. L. Liu sp. nov. Figure 3

**FIGURE 3:**
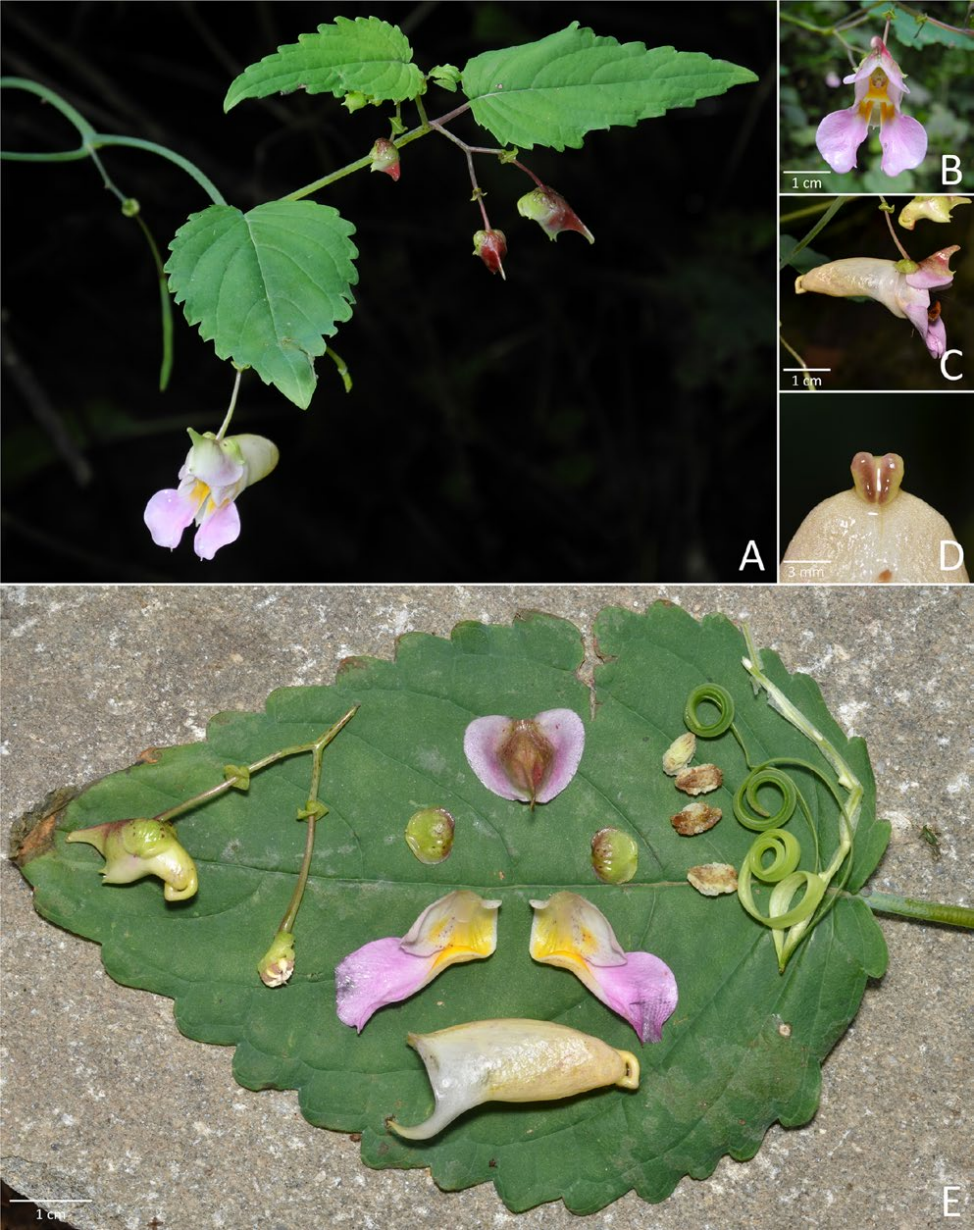
*Impatiens perforata* sp. nov.: (A) habit, (B) front view of the corolla, (C) side view of the corolla, (D) closer look at the spur, (E) inflorescence, dehiscent fruit (showing seeds) and dissection of the corolla.

*Type*. CHINA. Gansu Province, Longnan City, Wen County, Danbao Township, Shangba Village to Bimagou Village, 1600 m, 02 September 2013, stream valley, hillside, forest margin, *Zai‐Min Jiang* *et al. 3687* (holotype: WUK!; isotypes: WUK!).

*Diagnosis*. *Impatiens perforata* resembles *I*. *compta* by lower sepal pale yellow, saccate; spur incurved, but differs in bracts cupuliform, amplexicaul (vs. lanceolate); lower sepal abruptly constricted into an extremely short, incurved, bifid spur (vs. abruptly narrowed into an incurved spur, ca. 1 cm); upper petal abaxial midvein with long and broad rostellum at middle (vs. apex long rostellate). It is also allied to *I*. *uncata* by lower sepal pale yellow, saccate; dorsal sepal and lateral united petals pink; spur short, incurved, bifid, but differs in bracts cupuliform, amplexicaul (vs. linear‐lanceolate); dorsal petal abaxial midvein with long and broad rostellum at middle (vs. abaxial midvein thickened, cristate at middle); lateral united petals lower lobes elliptic (vs. narrowly lorate).

*Description*. Annual herb, up to 70 cm tall. Stems erect. Leaves alternate; petioles 1.5–5.5 cm long; lamina lanceolate to ovate, glabrous, apex acute, base subcordate to rounded, margin crenate; lateral veins 3–5 pairs. Inflorescences in upper leaf axils, descending, 1–3‐flowered; peduncles 1–2.5 cm long; pedicels 1.5–2.5 cm long, with bract at middle, elongated at fruiting; bract persistent, cupuliform, amplexicaul, 3–3.5 × 3–4 mm, apex acute. Flower pink, 4–5 cm long. Lateral sepals 2, orbicular, 5–6 × 5–6 mm, abaxial midvein fine, apex mucronate. Lower sepal deeply saccate, red spotted, 2.5–3.5 cm deep excluding spur; mouth flat, 1–1.5 cm wide, with a protrusion ca. 6 mm long; abruptly constricted into an extremely short, incurved, bifid spur, 4–5 mm long. Dorsal petal 1–1.5 × 1–1.5 cm, ovate, apex retuse; abaxial midvein with long and broad rostellum at middle, ca. 6 mm long. Lateral united petals not clawed, 2.5–3 cm long, two‐lobed, with yellow patch and red spots at base; lower lobes elliptic, ca. 6 mm wide, apex rounded; upper lobes broadly dolabriform, ca. 11 mm wide, margin entire. Stamens with yellow anthers; anther oblong, apex acute. Ovary linear, shorter than stamens. Fruit a linear capsule, 4.5–6 × 0.25–0.3 cm, apex beaked. Seeds ovoid, surface carinate.

*Phenology*. Flowering and fruiting from August to September.

*Etymology*. The specific epithet “*perforata*” refers to the cupuliform, amplexicaul bracts of the new species. The proposed Chinese vernacular name is “穿苞凤仙花”.

*Additional specimen examined (paratypes)*. CHINA. Gansu Province, Longnan City, Wen County, Danbaohe Protection Station, Yangshanhe to Bimagou. 104°33′55″ E, 32°51′59″ N, 1500 m, 09 Aug 2010, roadside under forest, *Zai‐Min Jiang* *et al. 2024* (WUK!).

*Distribution and habitat*. This species was only found in Wen County, Gansu Province. It grows on hillside under forest at 1500–1600 m.

### 5.2 *Impatiens aciformis* Z. J. Huang & P. L. Liu sp. nov. Figure 4; Plate 211 in Ho (1981)

**FIGURE 4:**
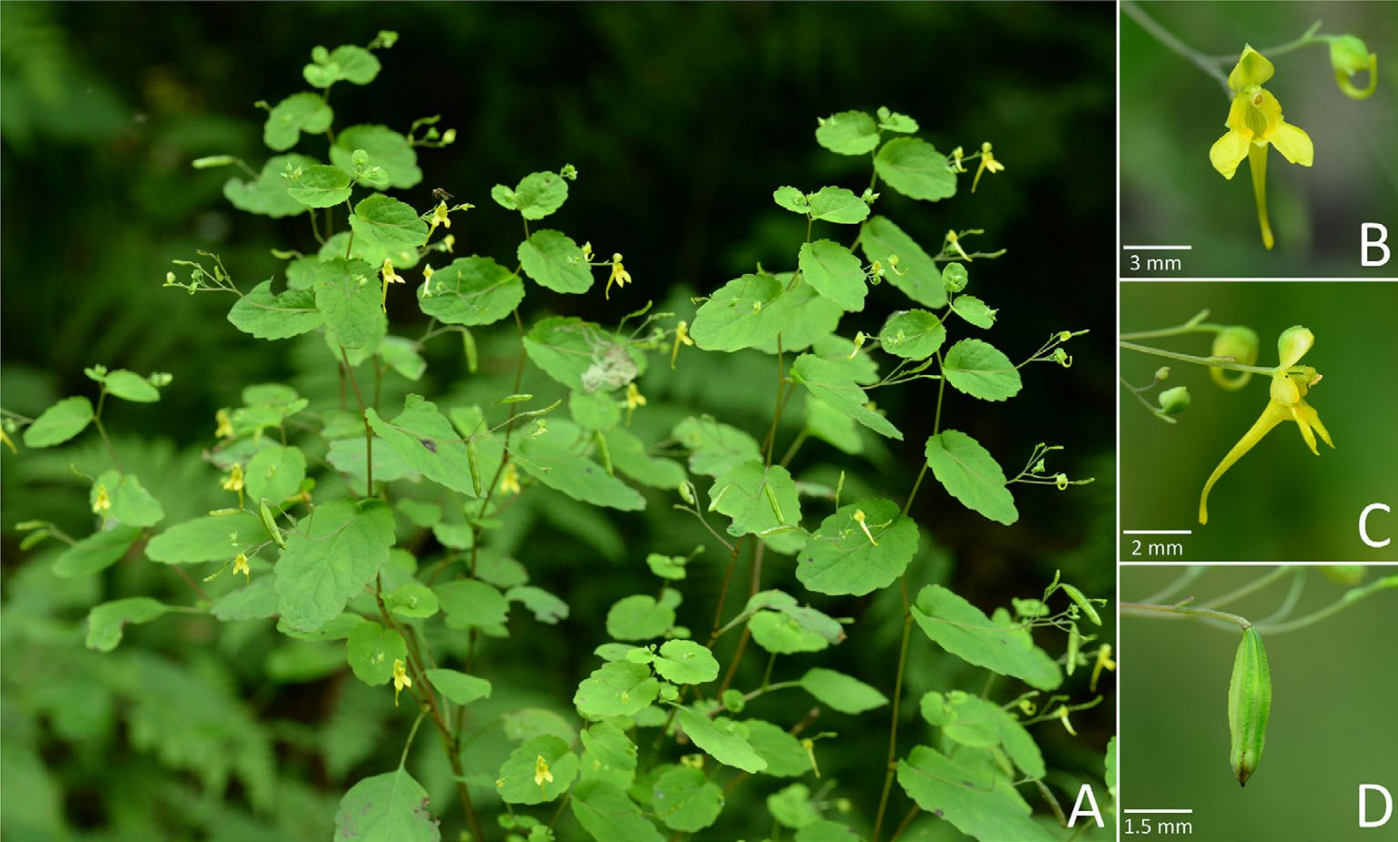
*Impatiens aciformis* sp. nov.: (A) habit, (B) front view of the corolla, (C) side view of the corolla, (D) fruit.

*Type*. CHINA. Shaanxi Province, Baoji City, Feng County, Xinjiashan Forestry Station, Tongtianhe National Forest Park, 106°37′6″ E, 34°9′17″ N, 1380 m, 15 August 2011, *Zai‐Min Jiang* *et al. 2983* (holotype: WUK!; isotypes: WUK!).

**FIGURE A4:**
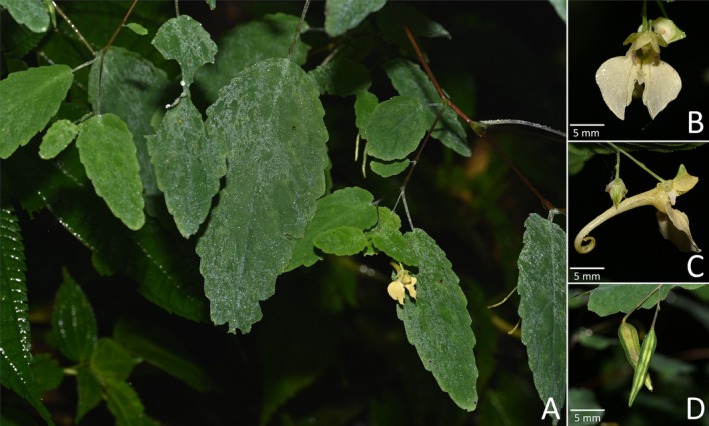
*Impatiens undulata*: (A) habit, (B) front view of the corolla, (C) side view of the corolla, (D) fruit.

**FIGURE A5:**
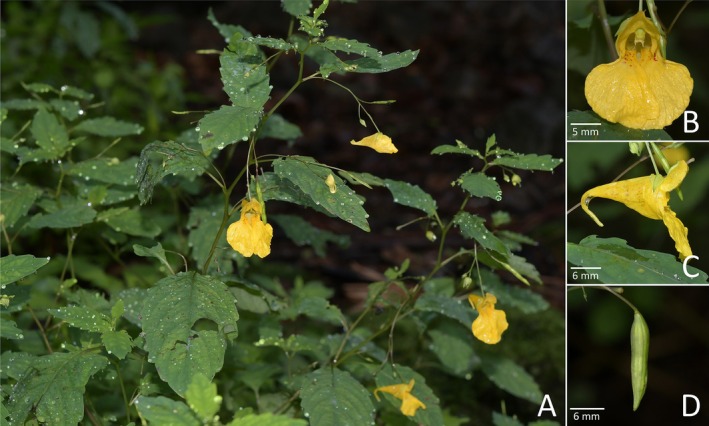
*Impatiens noli‐tangere*: (A) habit, (B) front view of the corolla, (C) side view of the corolla, (D) fruit.

**FIGURE A7:**
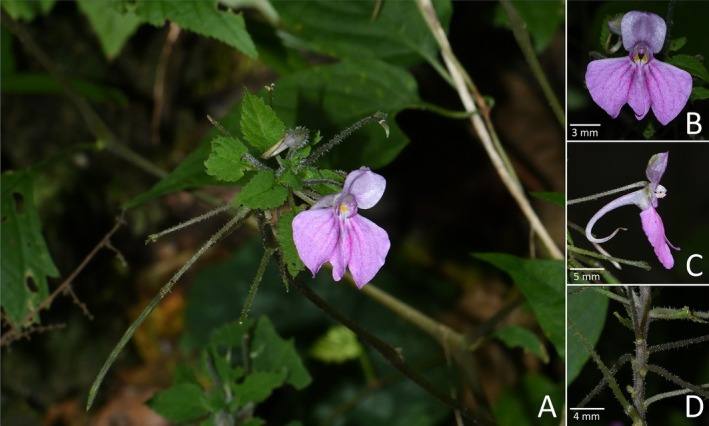
*Impatiens dasyvexilla*: (A) habit, (B) front view of the corolla, (C) side view of the corolla, (D) closer look of the stem, showing the indumentum.

**FIGURE A11:**
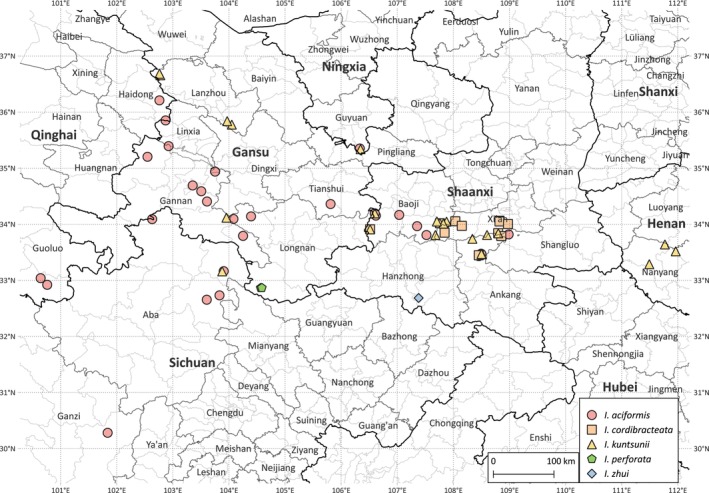
Distribution map of new species.

*Diagnosis*. *Impatiens aciformis* is akin to *I*. *notolopha* by lamina margin crenate; corolla yellow, but is distinguishable by lamina ovate to suborbicular, base subcordate to cordate; corolla, lateral united petals and spur shorter than 1 cm; the ratio of spur and lateral united petals > 1 (vs. lamina broadly ovate or ovate‐elliptic, base broadly cuneate, abruptly attenuate into petiole; corolla, lateral united petals and spur longer than 1 cm; the ratio of spur and lateral united petals ca. 1:1). It also bears resemblance to *I. undulata* (Figure A4) by lamina margin crenate; corolla yellow, but differs in corolla, lateral united petals and spur shorter than 1 cm; dorsal petal abaxial midvein slightly carinate (vs. corolla, lateral united petals and spur longer than 1 cm; dorsal petal abaxial midvein broadly carinate).

*Description*. Annual herb, up to 50 cm tall. Stem erect, reddish, well branched. Leaves alternate; petioles 0.6–3 cm long, slender, becoming shorter upward; lamina ovate to suborbicular, 1–4.5 × 1–2.5 cm, glabrous, apex obtuse to rounded to truncate, base subcordate to cordate, auriculate to amplexicaul, margin crenate; lateral veins 3–5 pairs. Inflorescences in upper leaf axils, ascending, 2–5 (−8)‐flowered; peduncle 2–4 cm long, longer than petioles, pedicels 0.2–0.5 cm long, slender, basally bracteate, elongated at fruiting; bract persistent, linear, 1–1.5 mm long, apex obtuse. Flower yellow or pale yellow, 6–8 mm long. Lateral sepals, asymmetrically narrowly ovate, 1.5–2 × 1–1.3 mm, abaxial midvein thickened, apex obtuse. Lower sepal narrowly infundibuliform or cornute, without red spots, 0.8–1 mm deep excluding spur; mouth flat, 2.5–3.5 mm wide; spur entire, straight to slightly curved, 3.5–6.5 mm long. Dorsal petal 2–2.5 × 0.9–1.5 mm, ovate to suborbicular, apex mucronate, abaxial midvein slightly carinate. Lateral united petals not clawed, 3.5–5 mm long, two‐lobed, with or without red spots at base; lower lobes oblong, ca. 1 mm wide, apex rounded; upper lobes broadly dolabriform, ca. 1.5 mm wide, margin notched. Stamens with yellow anthers, anthers pyramidal‐ovoid, apex acute. Ovary linear, shorter than stamens. Fruit a linear capsule, 2.5–3.5 × 0.3–0.5 mm, apex beaked. Seeds ovoid, surface colliculate.

*Phenology*. Flowering from July to October, fruiting from August to October.

*Etymology*. The specific epithet “*aciformis*” refers to the tiny, acerate spur of the new species. The proposed Chinese vernacular name is “针距凤仙花”.

*Additional specimen examined (paratypes)*. CHINA. Shaanxi: Feng County, Huangniupu Town, Matoutan Forestry Station, Dawangshan, 2500 m, under forest, 27 July 2008, *Zai‐Min Jiang 266* (WUK!); Ningshan County, Pingheliang, National Highway 210 (1079–1085 km), 2200 m, 25 August 2009, *Zai‐Min Jiang 1316* (WUK!); Feng County, Xinjiashan, 2200 m, mountainous area, mountain top, roadside, 18 July 1964, *Kun‐Tsun Fu 16072* (WUK!); Ningshan County, Pingheliang, 2200 m, 30 July 1978, *Kun‐Tsun Fu 17922* (WUK!); Feng County, Xinjiashan, Beigoutan, 2200 m, mountainous area, mountain top, roadside, 18 July 1964, *Kun‐Tsun Fu 16120* (WUK!); Feng County, Xinjiashan, 2200 m, mountainous area, mountain top, roadside, 18 July 1964, *Kun‐Tsun Fu 16072* (WUK!); (Mei County) Taibaishan, South slope, 2300 m, valley, stream side, in grassland, 07 September 1957, *Guo‐Xian Li 1715* (WUK!); Ningshan County, Huoditang, Pingheliang, 2400 m, under forest, 17 July 2008, *Zai‐Min Jiang* *et al. 95* (WUK!); Taibai County, Huangbaiyuan, Taiyang Highway, 2200–2950 m, Aoshan south trailhead, 12 August 2011, *Zai‐ Min Jiang et al.* *2832* (WUK!); Zhashui County, Yingpan Forestry Station, Chenjiagou, 1700 m, hillside, 19 June 1973, *Xi‐Xiang Hou* *et al. 980* (WUK!. IBSC0184405!); Taibai County, Dalingzi, hillside, under dense forest, 2468 m, 10 July 2011, *Si‐Feng Li* *et al. 15653* (XBGH!); Zhashui County, Yingpan Town, Huanghualing, hillside, under open forest, 1457 m, 24 July 2011, *Si‐Feng Li 15881* (XBGH!).

Gansu: Tianshui City, Dangchuan Town, Huoyanshan, 2200 m, valley, under forest, 07 August 1963, *Pung‐Chao Kuo 4961* (WUK!); Zhouqu County, Shatan Forestry Station, 2400 m, hillside, under forest, 27 June 1964, *Pung‐Chao Kuo 5145* (WUK!); Zhouqu County, Shatan Forestry Station, 2300 m, riverbank, 29 June 1964, *Pung‐Chao Kuo 5219* (WUK!); Lamkungpa, 3600 m, on wet place in valley, 02 September 1937, *Kun‐Tsun Fu 1617* (WUK!, PE00077632!); Near Chuoni, 2900 m, in valley, often under bushes, 24 July 1936, *T. P. Wang 5338* (WUK!, PE00077624!); Ling‐Tan‐hsien, Kan‐Ko, 2600 m, 22 September 1940, *W.Y.Hsia 8706* (WUK!, PE00077633!); Hsiahohsien, Chingshui, 2500 m, on shady and wet place, *Kun‐Tsun Fu 1044* (WUK!, PE00077625!); Near Minshan, Lamkungpa, 3700 m, in valley, 03 September 1957, *Wang Tso‐Pin 7662* (WUK!, PE00077629!); Tanchang County, Lijiagou, 2000 m, valley, under bushes, 11 August 1985, *Huangtudui 6198* (WUK!); Zhuomin County, Dayugou, 2700 m, under hillside, 09 August 1985, *Huangtudui 6085* (WUK!); Lintan County, Lianhuashan, 3000 m, under forest, 09 August 2020, *Pei‐Liang Liu 919* (WUK!); Tanchang County, 2613 m, forest edge, 02 August 2020, *Pei‐Liang Liu 861* (WUK!).

Qinghai: Banma County, Makehe woodland, Gerizegou, 3600 m, floodplain of tributary ditch, under fir forest, *Shi Chen 60117* (WUK!); Banma County, Makehe woodland, Yadaiyin to De'ang, 3800 m, under forest, ridge, in crevices, 18 July 1960, *Shi Chen 60015* (WUK!); Minhe County, Xing'ergou, 2240 m, 23 September 1964, *Shi Chen 2438* (WUK!); Minhe County Bazhou people's commune, Xigou, 2500 m, in ravine, 19 September 1959, *Qinggandui 1885* (WUK!).

Sichuan: Vicinity of Sungpan, 3200 m, on a wet and shady place, 20 September 1937, *Kun‐Tsun Fu 1862* (WUK!, PE01878441!); Kangding, 3240 m, forest edge, 31 July 2022, *Pei‐Liang Liu 1376* (WUK!); Jiuzhai Valley, Wuhuahai, 2356 m, under forest, 07 August 2020, *Pei‐Liang Liu 913* (WUK!); Songpan County, Huanglong, Huanglongzhongsi, 3462 m, forest edge, roadside, in grass, 06 August 2020, *Pei‐Liang Liu 909* (WUK!).

Ningxia: Jingyuan County, Erlongdong, 1800 m, hillside, grassland, 03 October 1984, *Jin‐Xiang Yang 5608* (WUK!).

*Distribution and habitat*. *Impatiens aciformis* is a widespread species. It also occurs in Gansu, Ningxia, Qinghai, and Sichuan besides the type locality in Shaanxi. It grows in shady places under bushes or in valleys at 1500–3700 m.

### 5.3 *Impatiens zhui* Z. J. Huang & P. L. Liu sp. nov. Figure 5

**FIGURE 5:**
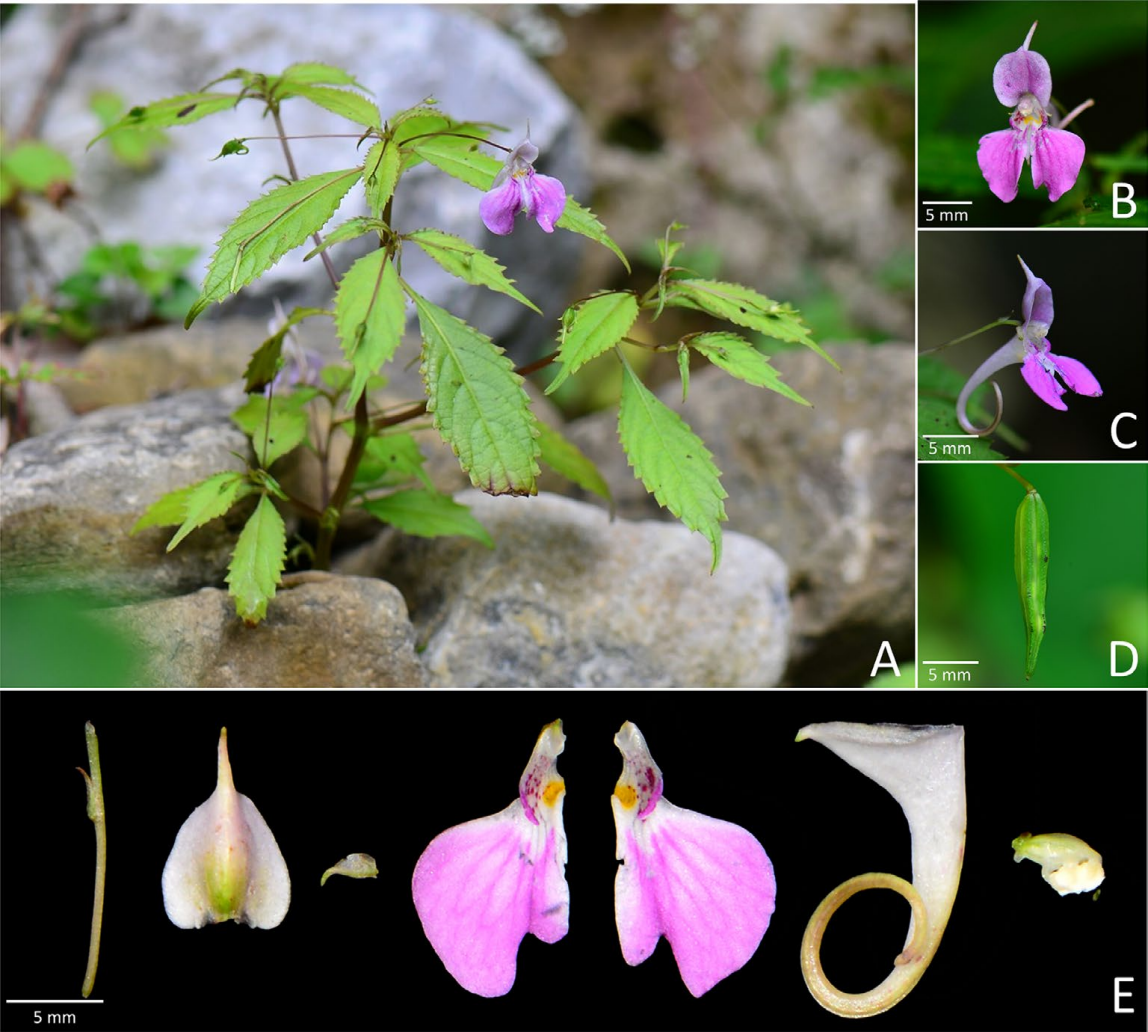
*Impatiens zhui* sp. nov.: (A) habit, (B) front view of the corolla, (C) side view of the corolla, (D) fruit, (E) dissection of the corolla. Photos by Ren‐Bin Zhu.

*Type*. CHINA. Shaanxi Province, Hanzhong City, Xixiang County, Hexi Township, 107°22′51.19″ E, 32°41′11.96″ N, 1023 m, 12 October 2023, roadside in front of rock wall, *Pei‐Liang Liu 1631* (holotype: WUK!, isotypes: WUK!).

*Diagnosis*. *Impatiens zhui* resembles *I*. *pterosepala* by corolla pink; lower sepal white, narrowly infundibuliform; dorsal petal suborbicular; lateral united petals not clawed, with orange yellow patches and red spots at base, lower lobes orbicular, apex rounded, upper lobes broadly dolabriform, margin entire, but differs in top of dorsal petal abaxial midvein carinate, near the top of the crest caudate (vs. dorsal petal abaxial midvein thickened). It is also similar to *I*. *dasyvexilla* by corolla pink; lower sepal white, narrowly infundibuliform; dorsal petal suborbicular; lateral united petals not clawed, with orange yellow patches and red spots at base, lower lobes orbicular, apex rounded, upper lobes broadly dolabriform, margin entire, but can be differentiated by whole plant glabrous; dorsal petal abaxial midvein carinate, near the top of the crest caudate (vs. whole plant densely pubescent or pilose; abaxial midvein with a narrow wing, apex of wing rounded).

*Description*. Annual herb, up to 80 cm tall. Stem erect, well branched even from the base. Leaves alternate; petioles 1–2.5 cm long, becoming shorter upward; lamina lanceolate to ovate, 6– 9(−12) × 2–5 cm, glabrous, apex acute, base attenuate, margin serrate to crenate, teeth apex mucronate; lateral veins 5–7 pairs. Inflorescences in upper leaf axils, ascending, 1‐flowered; peduncle 1.5–2.5 cm long, pedicels ca. 1 cm long, slender, with bract at almost the top, elongated at fruiting; bract persistent, linear, 2.5–3 mm long, apex acute. Flower pink, 2.5–3 cm long. Lateral sepals 2, asymmetrically ovate, 2.5–3 × 1–1.5 mm, abaxial midvein thickened, apex acuminate. Lower sepal white, narrowly infundibuliform, 3–4 mm deep excluding spur; mouth flat, 7–9 mm wide; spur entire, incurved, 1.5–2 cm long. Dorsal petal 5–6 × 5–6 mm, suborbicular, apex acuminate, abaxial midvein carinate, near the top of the crest caudate. Lateral united petals not clawed, 1.3–1.5 cm long, two‐lobed, with orange yellow patches and red spots at base; lower lobes orbicular, ca. 1.7 mm wide, apex rounded; upper lobes broadly dolabriform, ca. 6 mm wide, margin entire. Stamens with pale yellow anthers, anthers pyramidal‐ovoid, apex acute. Ovary linear, shorter than stamens. Fruit a linear capsule, 2.5–3 × 0.4–0.5 cm, apex acute. Seed ovoid, surface colliculate.

*Phenology*. Flowering from June to October, fruiting from July to November.

*Etymology*. The species epithet “*zhui*” is named after Dr. Ren‐Bin Zhu from Xishuangbanna Tropical Botanical Garden of Chinese Academy of Sciences (XTBG, CAS), who first photographed this species in 2016. The proposed Chinese vernacular name is “仁斌凤仙花”.

*Additional specimen examined (paratypes)*. CHINA. Shaanxi: Hanzhong City, Xixiang County, Hexi Township, 107°24′29.92″ E, 32°39′5.36″ N, 945 m, 12 Oct. 2023, roadside in front of rock wall, *Pei‐Liang Liu 1630* (WNU!).

*Distribution and habitat*. *Impatiens zhui* is only known from its type locality Xixiang County. It grows by roadsides near streams at 950–1050 m.

### 5.4 *Impatiens kuntsunii* Z. J. Huang & P. L. Liu sp. nov. Figures 6 and 7

**FIGURE 6:**
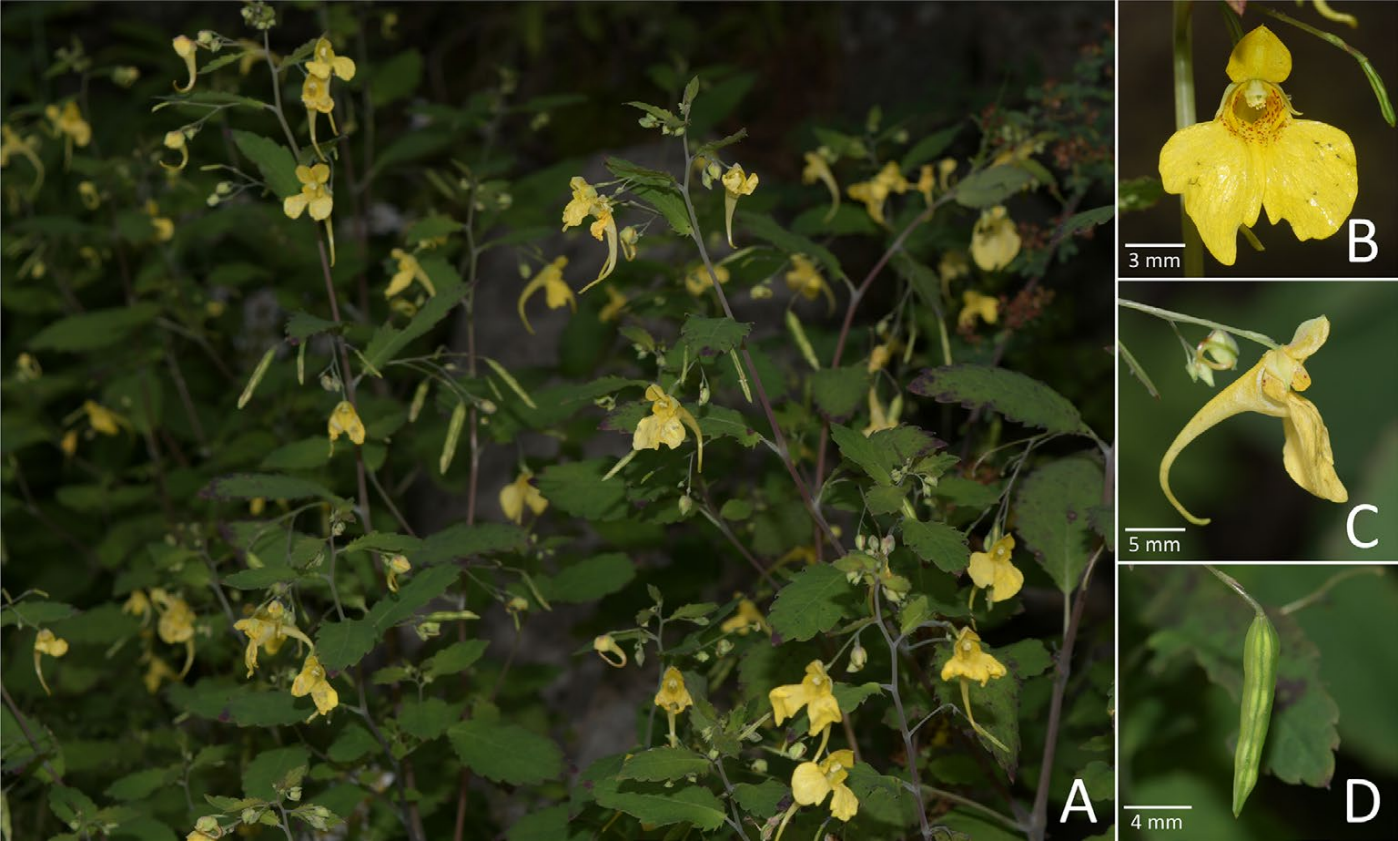
*Impatiens kuntsunii* sp. nov.: (A) habit, (B) front view of the corolla, (C) side view of the corolla, (D) fruit.

**FIGURE 7:**
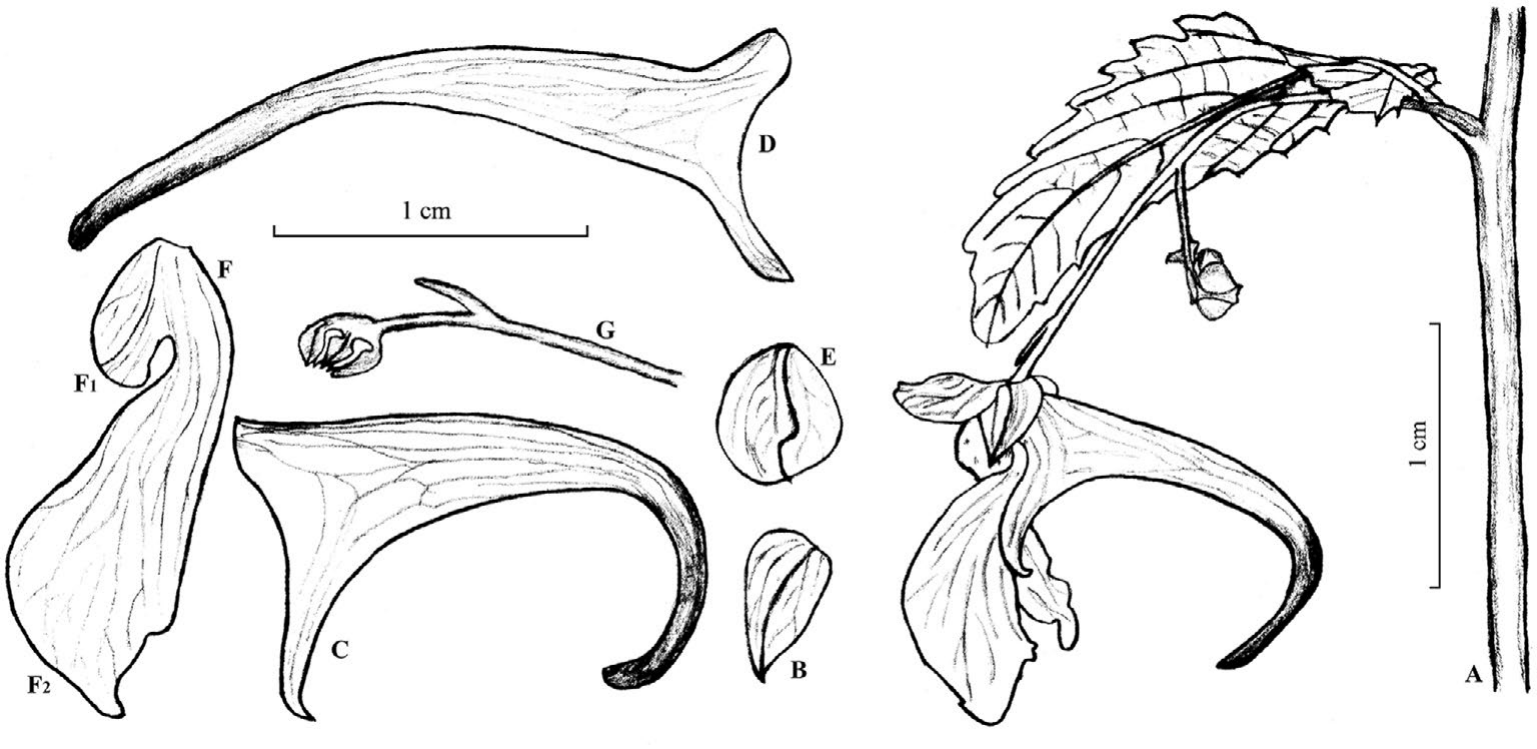
Line drawings of *Impatiens kuntsunii* sp. nov.: (A) a descending inflorescence in leaf axil, (B) lateral sepal, (C, D) lower sepal, (E) dorsal petal, (F) lateral united petal, F1 lower lobe, F2 upper lobe, (G) pedicle, bract and androecium. Drawn by Pei‐Liang Liu, A–C, E–G based on *Zai‐Min Jiang* *et al. 1222* (WUK), D based on *Zai‐Min Jiang* *et al. 1567* (WUK).

*Type*. CHINA. Shaanxi Province, Ankang City, Ningshan County, Pingheliang 108°30′37″ E, 33°27′48″ N, streamside on forest margin, 2100 m, 21 August 2009, *Zai‐Min Jiang* *et al. 1222* (holotype: WUK!; isotypes: WUK!).

*Diagnosis*. *Impatiens kuntsunii* resembles *I. noli‐tangere* by lamina elliptic to ovate, margin serrate; corolla yellow, but can be identified by lower sepal narrowly infundibuliform, without red spots (vs. broadly infundibuliform, with red spots); apex of the lower lobe of lateral united petals obtuse, gradually widening from base to apex (vs. apex rounded, gradually tapering from base to apex). It is also similar to *I*. *notolopha* by lamina elliptic to ovate; corolla yellow, but differs in lamina margin serrate, teeth apex mucronate (vs. crenate, teeth apex rounded or retuse); inflorescence descending (vs. ascending); lateral sepals asymmetrically narrowly ovate (vs. broadly ovate); lower lobe of lateral united petals oblong, ca. 2 mm wide (vs. suborbicular, small).

*Description*. Annual herb, to 100 cm tall. Stems erect, succulent, reddish, well branched, with swollen nodes in the lower part. Leaves alternate; petioles 1–5 cm long, slender, becoming shorter upward; lamina elliptic to ovate, 2.5–6 (−9.5) × 1–3 (−4) cm, glabrous, apex acute and mucronate, base cuneate or almost rounded in upper leaves, margin serrate, with 10–15 (−19) teeth on each side of leaf, teeth acute and always mucronulate at apex, lower teeth smaller and often with longer mucrones; lateral veins 5–9 pairs. Inflorescences in upper leaf axils, descending, 3–5‐flowered; peduncle 1–1.5 cm long, longer than petioles, pedicels 0.3–0.5 cm long, slender, 1‐bracteate in upper part, elongated at fruiting; bract persistent, linear‐lanceolate, 1.5–3 mm long, apex acute. Flower yellow or pale yellow, 1.5–2 cm long. Lateral sepals 2, asymmetrically narrowly ovate, 3–5 × 1.5–3 mm, abaxial midvein thickened, apex mucronate. Lower sepal narrowly infundibuliform or cornute, without red spots, 0.3–0.5 cm deep excluding spur; mouth flat, 0.6–1 cm wide, asymmetrically emarginate; spur entire, rarely bifid, slightly incurved or almost straight, 0.8–1 cm long. Dorsal petal 4–5 × 4–4.5 mm, orbicular, apex mucronate, abaxial midvein slightly carinate. Lateral united petals not clawed, 1.2–1.7 cm long, two‐lobed, with or without red spots at base; lower lobes oblong, ca. 2 mm wide, widen toward the apex, apex truncate; upper lobes broadly dolabriform, 5–6 mm wide, margin undulate, notched or rarely entire. Stamens with yellow anthers, anthers pyramidal‐ovoid, apex acute. Ovary linear, shorter than stamens. Fruit a linear capsule, 1–2 × 0.2–0.3 cm, apex beaked. Seeds ellipsoid, 2.5–3.5 × 1–1.5 mm, brown, with ribs and protuberances on surface.

*Phenology*. Flowering from July to September, fruiting from August to October.

*Etymology*. The epithet “*kuntsunii*” highlights our homage to Prof. Kun‐Tsun Fu (1912–2010), a phytotaxonomist from Northwestern Institute of Botany and Northwest A&F University, who devoted all his life to the study of plant diversity in northwestern China, especially in the Qinling–Bashan Mountains. The proposed Chinese vernacular name is “坤俊凤仙花”.

*Additional specimen examined (paratypes)*. CHINA. Shaanxi: (Mei County) Taibaishan, Lotosze (Luotuosi), 2100 m, 6 August 1933, *T. P. Wang 1886* (WUK!, PE00077618!); Mei County, Taibaishan, near Dadian, 2300 m, 29 August 1937, *T. N. Liou and P. C. Tsoong 385* (WUK!, PE00077615!); same details but *434* (WUK!, PE00077617!); Hu County, Laoyu, Zhuque, along road, 2250 m, 19 August 2002, *Xizhi‐Caijidui 02149* (WUK!); Ningshan County, Pingheliang, 1850 m, 25 August 2009, *Zai‐Min Jiang* *et al.* *1314* (WUK!); Ningshan County, Caiziping, 2000 to 2100 m, 30 July 2010, *Zai‐Min Jiang* *et al.* *1567* (WUK!); Feng County, Matoutan Linchang, forest margin, 2400 m, 22 August 2012, *Zai‐Min Jiang* *et al.* *3655* (WUK!); Mei County, Taibaishan, Honghegu shihai to Xiabansi, hillside, shady place, 2341 m, 28 July 2009, *Si‐Feng Li* *et al.* *12077* (XBGH!); (Mei County), Taibaishan, Dadian, 2400 m, 31 July 2012, *Zhao‐Yang Chang 2012184* (WUK!); Mei County, Taibaishan, Honghuaping to Xiabansi, 2500 m, forest edge, 10 August 2021, *Pei‐Liang Liu 1321* (WUK!); Xi'an City, Chang'an District, Fengyu, Fenshuiling, 2050 m, forest edge, roadside, 02 August 2018, *Pei‐Liang Liu 424* (WUK!); Feng County, Xinjiashan Forestry Station, Tongtianhe National Forest Park, 1000 to 2000 m, 15 August 2011, *Zai‐Min Jiang* *et al. 2976* (WUK!).

Gansu: (Yongdeng County) Liancheng, Tianwang Gou, in valley, 2300 m, 2 September 1960, *Qinggandui 3423* (WUK!, PE00078939!); Yongdeng County, Liancheng, Datulugou, Pubugou, 36° 42.559′ N, 102° 39.955′ E, 2790 m, 10 August 2006, *Xue‐Gang Sun* *et al. 0653* *8* (PE01832361!); Yuzhong County, Mount Xinglongshan, near spring, 2200 m, 27 August 1982, *Zhao‐Ying Yu & Yang‐Peng Xu* *3728* (WUK!); Diebu County, Lazikou Town, Jiulicai Village, Hougou, 2353 m, by river, grassland, 2 August 2020, *Pei‐Liang Liu 869* (WUK!); Lanzhou City, Yuzhong County, Xinglongshan, 2350 m, forest edge, roadside, 26 July 2024, *Pei‐Liang Liu 1652* (WUK!).

Ningxia: Jingyuan County, Daxueshan Linchang, in valley, 2100 m, 4 July 2012, *Ren‐Bin Zhu 140* (WUK!).

Henan: Nanzhao, Baotianman, in valley, 1170 m, 12 August 1963, *Sui‐Yi Wang 632018* (HEAC!); Nanzhao, Qiaoduan, in valley, 1000 m, 28 July 1964, *Sui‐Yi Wang* *641211* (HEAC!); Xixia, Heiyanzhen, in forest, 1200 m, 3 August 1960, *Sui‐Yi Wang* *605524* (HEAC!); Xixia, Laojieling, in forest, 1200 m, 17 August 1991, *Yong‐Zhong Ye 91589* (HEAC!).

Sichuan: Jiuzhai Valley, Wuhuahai, 2465 m, under forest, 07 August 2020, *Pei‐Liang Liu 912* (WUK!).

*Distribution and habitat*. Unlike many species of *Impatiens* that have very restricted distributions, *I*. *kuntsunii* has a relatively broader range of distribution. Besides the type locality (Ningshan County, Shaanxi) and the neighboring county of Hu County, Mei County, and Feng County, *I*. *kuntsunii* is also found in central Gansu (Yongdeng County and Yuzhong County), southern Ningxia (Jingyuan County), western Henan (Nanzhao County and Xixia County) and northeast Sichuan (Jiuzhaigou County). It grows in montane regions, forests, forest fringes, streamsides, and moist grasslands, at 1000–2790 m.

### 5.5 *Impatiens cordibracteata* Z. J. Huang & P. L. Liu sp. nov. Figure 8

**FIGURE 8:**
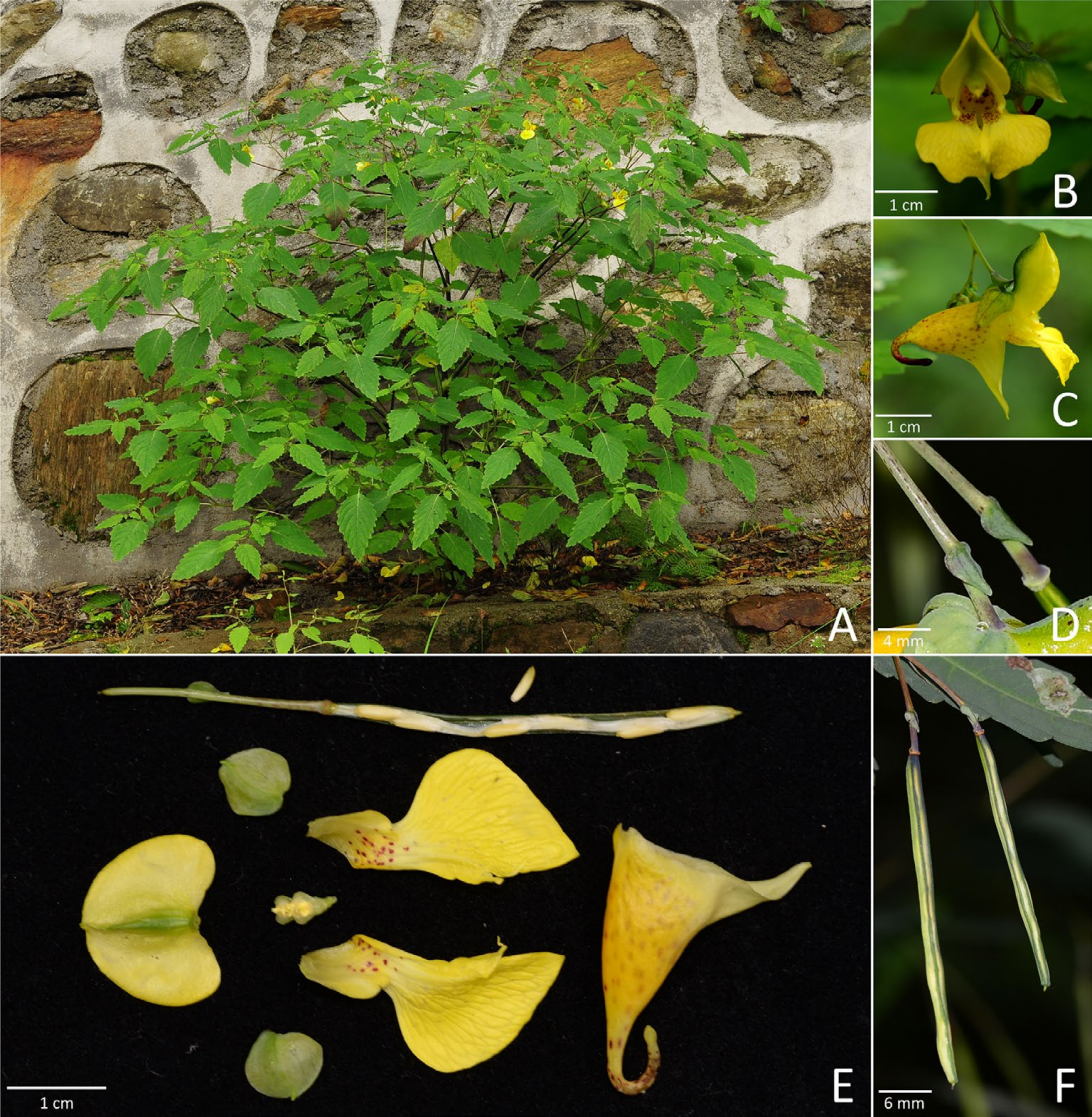
*Impatiens cordibracteata* sp. nov.: (A) habit, (B) front view of the corolla, (C) side view of the corolla, (D) closer look of the bract, (E) dehiscent fruit (showing seeds) and dissection of the corolla, (F) fruits.

*Type*. CHINA. Shaanxi Province, Xi'an City, Chang'an District, Fengyu, Fenshuiling, 108°47′54″ E, 33°50′32.66″ N, 2171 m, 02 August 2018, roadside on forest edge, *Pei‐Liang Liu 425* (holotype: WUK!, isotypes: WUK!, WNU!).

*Diagnosis*. *Impatiens cordibracteata* is similar to *I*. *latebracteata*, *I*. *sutchuenensis*, and *I*. *longialata* by corolla yellow, lower sepal infundibuliform, but differs from *I*. *latebracteata* by bract ovate to broadly ovate, margin entire; lateral sepals symmetrical (vs. bract orbicular, margin dentate; lateral sepals asymmetrical). It can be differentiated from *I*. *sutchuenensis* by bract ovate to broadly ovate; lower sepal infundibuliform (vs. bract linear; lower sepal infundibuliform to slightly saccate). It also diverges from *I*. *longialata* by bract ovate to broadly ovate, 4.5–5.5 × 2.5–3.5 mm (vs. bract lanceolate to narrowly ovate, 2–3 mm long).

*Description*. Annual herb, up to 100 cm tall. Stem erect, well branched. Leaves alternate; petioles 0.5–3.5 cm long, becoming shorter upward; lamina broadly lanceolate to ovate, 4–8 × 2–5 cm, apex obtuse to slightly retuse, base rounded to subcordate, margin crenate; lateral veins 5–7 pairs. Inflorescences in upper leaf axils, descending, 2–5‐flowered; peduncles 4–4.5 cm long, pedicels 1–4 cm long, with bract at base, elongated at fruiting; bract persistent, ovate to broadly ovate, 4.5–5.5 × 2.5–3.5 mm, apex acute, margin entire. Flower yellow, 2.5–3.5 cm long. Lateral sepals 2, suborbicular to orbicular, 8–8.5 × 7–8 mm, abaxial midvein slightly thickened, apex mucronate. Lower sepal infundibuliform, with red spots, 1.8–2 cm deep excluding spur; mouth flat, 1.5–1.7 cm wide; spur entire, incurved, 7–9 mm long. Dorsal petal 0.9–1.5 × 0.9–1.5 cm, orbicular, apex mucronate, abaxial midvein slightly carinate. Lateral united petals not clawed, ca. 2 cm long, two‐lobed, with red spots at base; lower lobe orbicular or broadly oblong, ca. 4.5 mm wide, apex rounded; upper lobes broadly dolabriform, ca. 8 mm wide, margin entire. Stamens with yellow anthers, anthers pyramidal‐ovoid, apex acute. Ovary linear, shorter than stamens. Fruit a linear capsule, 2–4.5 × 0.2–0.3 cm, apex mucronate. Seeds ovoid, surface colliculate.

*Phenology*. Flowering from July to September, fruiting from August to October.

*Etymology*. The specific epithet “*cordibracteata*” refers to its large, entire, more or less heart‐shaped bracts of the new species. The proposed Chinese vernacular name is “心苞凤仙花”.

*Additional specimen examined (paratypes)*. CHINA. Shaanxi: Foping County, Houzhenzi Village, Damanghe, Luomadian, 1080 m, roadside, 13 September 1958, *Xiang‐Ming Zhang 560* (WUK!, KUN0429771!); Zhouzhi County, Chenhe Village, Erdaotan, 1320 m, roadside in valley, 26 August 1958, *Xiang‐Ming Zhang 251* (WUK!, KUN0429770!); Feng County, Matoutan Forestry Station, 1800 m, under forest, 22 August 2012, *Zai‐Min Jiang* *et al. 3653* (WUK!); Zhouzhi County, Taibeishan, Houzhingzhi, 900 m, by stream, 24 July 1999, *Zhu Chen and Xu Wang 1601* (KUN0429781!); Xi'an City, Chang'an District, Luan Town, Qinlingliang, 2038 m, hillside, under shrub, 07 August 2008, *Si‐Feng Li 11095* (XBGH!); Zhashui County, Yingpan Town, Huanghualing, 1457 m, hillside, under open forest, 24 July 2011, *Si‐Feng Li 15879* (XBGH!); Chang'an County, Fengyukou, Qinlingliang, 2083 m, hillside, under shrub, 24 July 2011, *Si‐Feng Li 15912* (XBGH!); Xi'an City, Chang'an District, Nanwutai, Liulantaogongguan, 1316 m, hillside, under open forest, 05 September 2008, *Si‐Feng Li 11662* (XBGH!); Mei County, Taibaishan, Honghegushihai to Xiabansi, 2234 m, valley, by water, 28 July 2009, *Si‐Feng Li 12121* (XBGH!); Ningshan County, Huoditang Forestry Station, 1500 m, near the station, 18 August 2009, *Zai‐Min Jiang* *et al. 1079* (WUK!).

*Distribution and habitat*. *Impatiens cordibracteata* occurs in the Qinling Mountains in Shaanxi. It grows under forest or on the forest edge at 900–2200 m.

## 6 New Records From the Qinling‐Bashan Mountains

### 6.1 *Impatiens cyanantha* Hook. f., Hooker's Icon. Pl. 29: t. 2866. 1908. Figure A6

*Type*. CHINA. Guizhou, September 1904, *J.H. Esquirol 226* (holotype E00313642).

*Specimens examined*. CHINA. Gansu: Wen County, Bikou Town, Dalaoling, 900 m, hillside, 23 October 1958, *Ye‐Chi Ho 1445* (WUK!); Wen County, Bikou District, Dadaoling, 1200 m, low hillside, 23 October 1958, *Zhi‐Ping Wei* *3322* (WUK!); Wen County, Bikou Town, Shilonggou, branch gully, 1000 m, 22 August 2011, *Zai‐Min Jiang* *et al. 3218* (WUK!); Wen County, Bikou Town, Shilonggou, Shuihaoping Village, 900 m, wet and shady place on the forest edge, 30 August 2013, *Zai‐Min Jiang* *et al. 3659* (WUK!); Wen County, Bikou Town, Shilonggou, Shuihaoping Village, 960 m, hillside under forest, 28 September 2024, *Pei‐Liang Liu* *1743* (WUK!).

### 6.2 *Impatiens dasyvexilla* Q. L. Gan & X. W. Li, Novon 28(4): 253. 2020. Figure A7

*Type*. China. Hubei: Zhuxi, Taoyuan Town, Duiwoping Village, Wuhuya, along the path near the forest margin, 31°42′35″ N, 109°50′13″ E, 1477 m, 24 September 2019, *X. W. Li 191721* (holotype HIB).

*Specimens examined*. CHINA. Shaanxi: Pingli County, Shizhai, Shuangping Town, 1000 m, hillside on rock, 12 July 1959, *Anonymous 362* (WUK!); Shanyang County, Tianzhushan, East slope, 1000 m, valley, by water, 26 September 1985, *Chao‐Ying Yu & Ying‐Peng Hsu 3977* (WUK!); Shanyang County, Tianzhushan, 800–1200 m, valley, by water, 26 September 1985, *Chao‐Ying Yu & Ying‐Peng Hsu 3949* (WUK!); Nanzheng County, Xishenba, wet and shady place, 11 October 1958, *Pei‐Yuan Li 528* (WUK!); Zhenping County, Muxihegou, 1300 m, valley, by water, under forest, 19 August 1990, *Guang‐Yuan Xu 5599* (WUK!); Zhenping County, Muxihe, 1313 m, roadside, 24 September 2024, *Pei‐Liang Liu 1734* (WUK!).

### 6.3 *Impatiens guiqingensis* S. X. Yu, Phytotaxa 247(3): 229. 2016. Figure A8

*Type*. CHINA. Gansu: Zhangxian County, Guiqingshan, shady places in forest, alt. 2440 m above sea level, 34°37′46″ N, 104°28′08″ E, 11 September 2008, *Cai‐Fei Zhang 1274* (holotype PE, isotype IBK).

*Specimens examined*. CHINA. Shaanxi: Feng County, Xinjiashan Forestry Station (Tongtianhe National Forest Park), 1600 m, under forest, 23 August 2012, *Zai‐Min Jiang 3652* (WUK!).

### 6.4 *Impatiens baishaensis* B. Ding & H. P. Deng, Phytotaxa 319(2): 193. 2017. Figure A9

Type. CHINA. Sichuan Province: Hongya County, Wawu Township, Baisha River, Wawu Shan Provincial Nature Reserve, 29°26′48.17″ N, 102°54′20.49″ E, ca. 2250 m a.s.l., 24 September 2015, *B. Ding 201,509060* (holotype PE, isotypes PE, SWNTU).

*Specimens examined*. CHINA. Shaanxi: Ankang City, Langao County, Taohe Commune, 1850 m, hillside, wet and shady place, 25 July 1959, *Pei‐Yuan Li 7756* (WUK!); Ningqiang County, Jiayan Town, Didonghe, 1158 m, in sinkhole, down the slope, 25 July 2021, *Pei‐Liang Liu 1270* (WUK!).

Sichuan: Mt. Omei, Jieyindian, 2514 m, under forest, 22 September 2023, *Pei‐Liang Liu 1613* (WUK!).

### 6.5 *Impatiens fargesii* Hook. f., Nouv. Arch. Mus. Hist. Nat. Sér. 4, 10: 256. 1908. Figure A10

*Type*. CHINA. Su‐tchuen oriental: District de Tchen Kéou Tin, s.d., *Farges s.n*. (P00780722!).

*Specimens examined*. CHINA. Shaanxi: Hanzhong City, Lueyang County, Guo Town to Jinjiahe, Xianggongshan, 1400 m, 30 July 2008, *Zai‐Min Jiang* et al. 539 (WUK!); Ankang City, Langao County, Taohe Commune, Heping Township, 1700 m, hillside, roadside, 27 July 1959, *Pei‐Yuan Li 7829* (WUK!); Pingli County, Qianjiaping, Sichahe, 1660 m, valley, under dense forest, 25 July 2006, *Yan‐Sheng Chen* *et al. 4202* (WUK!); Pingli County, Qianjiaping, Tianshuxia, 1589 m, roadside, 24 September 2024, *Pei‐Liang Liu 1736* (WUK!); Langao County, Qiancenghe, 1400 m, roadside, 25 September 2024, *Pei‐Liang Liu 1738* (WUK!).

Gansu: Wen County, Tielou Township, Baimahe Protection Station, 1500–1800 m, roadside, under forest, 07 August 2010, *Zai‐Min Jiang et al. 1653* (WUK!); Kang County, Qinghe Forestry Station, 1000–1500 m, 26 August 2011, *Zai‐Min Jiang et al. 3423* (WUK!); Wen County, Tielou Township, Baimahe Protection Station opposite, valley, 1625 m, shady and wet roadside, forest edge, 01 September 2013, *Zai‐Min Jiang et al. 3680* (WUK!).

## Author Contributions

**Zhang‐Jie Huang:** data curation (equal), formal analysis (equal), investigation (equal), methodology (equal), validation (equal), visualization (equal), writing – original draft (lead), writing – review and editing (equal). **Pei‐Liang Liu:** conceptualization (lead), formal analysis (equal), funding acquisition (lead), investigation (lead), methodology (lead), project administration (lead), resources (lead), supervision (lead), writing – original draft (equal), writing – review and editing (equal).

## Conflicts of Interest

The authors declare no conflicts of interest.

## Figures and Tables

**FIGURE A6:**
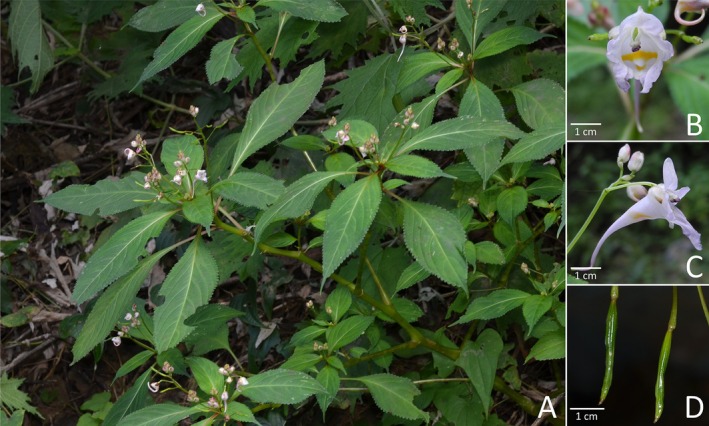
*Impatiens cyanantha*: (A) habit, (B) front view of the corolla, (C) side view of the corolla, (D) fruits.

**FIGURE A8:**
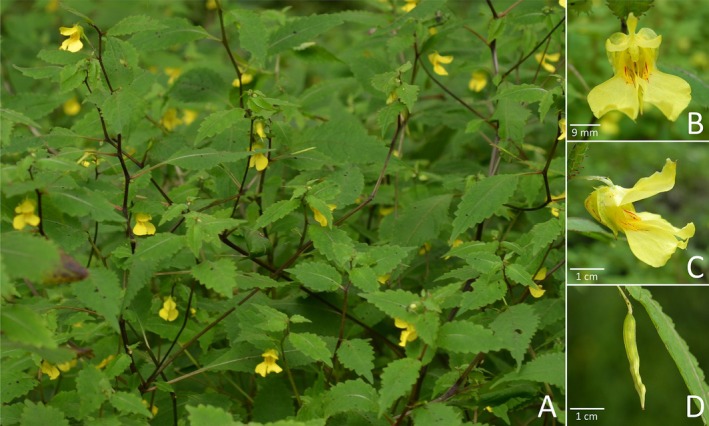
*Impatiens guiqingensis*: (A) habit, (B) front view of the corolla, (C) side view of the corolla, (D) fruit.

**FIGURE A9:**
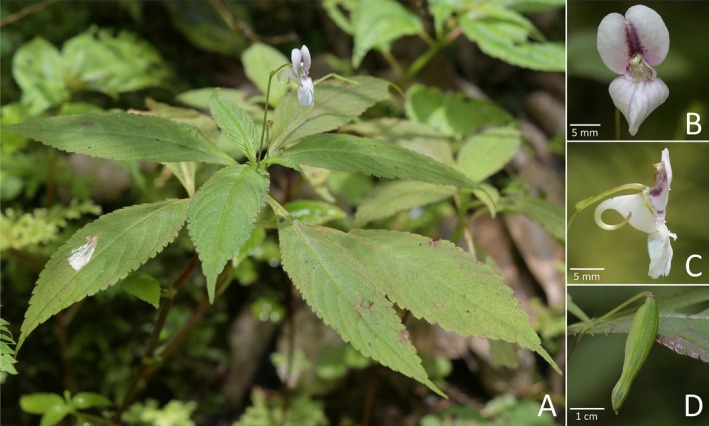
*Impatiens baishaensis*: (A) habit, (B) front view of the corolla, (C) side view of the corolla, (D) fruit.

**FIGURE A10:**
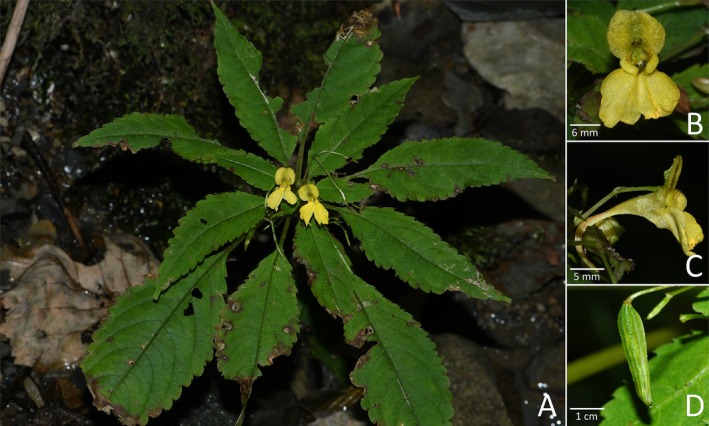
*Impatiens fargesii*: (A) habit, (B) front view of the corolla, (C) side view of the corolla, (D) fruit.

## Data Availability

The DNA sequences generated in the present study have been deposited in GenBank. The accession numbers and the information on the voucher specimens are available in Table A1. The voucher specimens of the new species are stored at WUK and WNU.

## Appendix A

**TABLE A1:** Species, locality, voucher information, and GenBank accession numbers for the sequences used in this study. Newly sequenced sequences were annotated as “*”.

Species	Locality	Voucher	GenBank accession number				Lab isolate No.
			ITS	*atpB*‐*rbcL*	*trnL*‐*F*	*psbA*‐*trnH*	
*Hydrocera triflora*	Hainan, China	HIB‐lzz18 (HIB)	—	MG162585			
*Hydrocera triflora*	Sri Lanka	Robyns 7260 0249369 (BR)	AY348853	—			
*Impatiens aciformis* sp. nov.*	Sichuan, China	Pei‐Liang Liu 1376 (WNU)	PX057458	PX067044	PX067113	PX067179	Q214
*Impatiens aciformis* sp. nov.*	Sichuan, China	Pei‐Liang Liu 913 (WNU)	PX057462	PX067048	PX067117	PX067183	Q218
*Impatiens aciformis* sp. nov.*	Gansu, China	Pei‐Liang Liu 919 (WNU)	PX057463	PX067049	PX067118	PX067184	Q219
*Impatiens aciformis* sp. nov.*	Gansu, China	Pei‐Liang Liu 861 (WNU)	PX057483	PX067069	PX067137	PX067204	Q244
*Impatiens aciformis* sp. nov.*	Sichuan, China	Pei‐Liang Liu 909 (WNU)	PX057484	PX067070	PX067138	PX067205	Q245
*Impatiens aciformis* sp. nov.*	Gansu, China	Zhao‐Yang Chang et al. 2013265 (WUK)	PX057498	PX067084	PX067151	PX067219	Q269
*Impatiens aciformis* sp. nov.*	Shaanxi, China	Zai‐Min Jiang et al. 266 (WUK)	PX057499	PX067085	PX067152	PX067220	Q270
*Impatiens aciformis* sp. nov.*	Shaanxi, China	Zai‐Min Jiang et al. 2983 (WUK)	PX057502	PX067089	PX067156	PX067224	Q275
*Impatiens alpicola*	Sichuan, China	LQE‐2017‐089 (QUA)	—	MK937571			
*Impatiens apsotis*	Sichuan, China	SX Yu et al. WS‐419 (PE)	—	OR135418			
*Impatiens aquatilis*	Yunnan, China	Song CNY017 (NEU)	AY348745	—			
*Impatiens arguta*	Yunnan, China	Yuan CN2k‐74 (NEU)	AY348746	—			
*Impatiens arguta*	Yunnan, China	Pan Li 008580	—	OP022554			
*Impatiens aurea*	North American origin, cult. Holden arboretum	L0388636 (L)	MH377175	—			
*Impatiens aurella*	—	—	SRR19405682				
*Impatiens baishaensis**	Shaanxi, China	Pei‐Liang Liu 1270 (WNU)	PX057468	PX067054	PX067123	PX067189	Q224
*Impatiens baishaensis**	Sichuan, China	Pei‐Liang Liu 1613 (WNU)	PX057471	PX067057	PX067126	PX067192	Q229
*Impatiens balansae*	Yunnan, China	S.X. Yu 4062 (PE)	KP776062	KP776012	KP776117	—	
*Impatiens balsamina*	Hunan, China	HF Wang 84 (PE)	—	OR135518			
*Impatiens balsamina*	cult. Guangzhou, China	Yuan CN2k1‐06 (NEU)	AY348749	—			
*Impatiens blinii*	Guangxi, China	S.X. Yu 4030 (PE)	KP776063	—			
*Impatiens bodinieri*	Guangxi, China	SX Yu 3715 (PE)	—	OR135496			
*Impatiens brachycentra*	central Asia	Burtt 3834 (E)	AY348756	—			
*Impatiens capensis*	Quebec, Canada	Küpfer s. n. (NEU)	AY348759	—			
*Impatiens capensis*	North Carolina, America	James S.Miller et al. 8923 (PE)	—	OR135466			
*Impatiens cavaleriei*	Guizhou, China	—	—	PQ156320			
*Impatiens chekiangensis*	Zhejiang, China	S.X. Yu et al. 20141	KP776064	KP776014	KP776121	—	
*Impatiens chimiliensis*	Yunnan, China	Yuan CN2k‐51 (NEU)	AY348760	—			
*Impatiens chiulungensis*	Sichuan, China	SX Yu 3989 (PE)	—	OR135488			
*Impatiens chiulungensis*	Sichuan, China	S.X. Yu et al. 3989 (PE)	KP776066	—			
*Impatiens chlorosepala*	Guangxi, China	S.X. Yu et al. 4209 (PE)	KP776067	—			
*Impatiens chlorosepala*	Guangxi, China	SWFU‐IBLE20161008	—	MW411293			
*Impatiens clavigera*	Guangxi, China	S.X. Yu 3717 (PE)	KP776068	KP776018	KP776125	—	
*Impatiens commelinoides*	Jiangxi, China	Southern Jiangxi Expedition Team 473 (PE)	—	OR135517			
*Impatiens compta*	Hubei, China	Lixw 1920360 (HIB)	MN685778	MN685776	MN685780	—	
*Impatiens compta*	Chongqing, China	B Ding BD201509041 (PE)	—	OR135438			
*Impatiens compta*	Hubei, China	SX Yu et al. 8339 (PE)	—	OR135470			
*Impatiens compta**	Shaanxi, China	Pei‐Liang Liu 1095 (WNU)	PX057477	PX067063	PX067131	PX067198	Q236
*Impatiens compta**	Shaanxi, China	Pei‐Liang Liu 1737 (WNU)	PX057518	PX067104	PX067171	PX067239	Q318
*Impatiens conaensis*	Xizang, China	FLPH 12‐0660 (PE)	—	OR135520			
*Impatiens conchibracteata*	Sichuan, China	Hao 427 (NEU)	AY348765	—			
*Impatiens conchibracteata*	Sichuan, China	LP150912	—	OP022555			
*Impatiens conchibracteata**	Sichuan, China	Pei‐Liang Liu 1628 (WNU)	PX057472	PX067058	PX067127	PX067193	Q230
*Impatiens corchorifolia*	Yunnan, China	SX Yu et al. 6403 (PE)	—	OR135477			
*Impatiens corchorifolia*	Yunnan, China	Chassot & Yuan 99‐173 (NEU)	AY348767	—			
*Impatiens cordibracteata* sp. nov.	Shaanxi, China	HN Qin et al. 19320 (PE)	—	OR135458			
*Impatiens cordibracteata* sp. nov.*	Shaanxi, China	Yuan Lu s.n. (WUK)	PX057459	PX067045	PX067114	PX067180	Q215
*Impatiens cordibracteata* sp. nov.*	Shaanxi, China	Pei‐Liang Liu 425 (WNU)	PX057476	PX067062	PX067130	PX067197	Q234
*Impatiens cordibracteata* sp. nov.*	Shaanxi, China	Zai‐Min Jiang et al. 1079 (WUK)	PX057508	PX067094	PX067161	PX067229	Q291
*Impatiens cornucopia*	Sichuan, China	SX Yu et al. 9858 (PE)	—	OR135464			
*Impatiens cornutisepala*	Guangxi, China	SX Yu 4023 (PE)	—	OR135487			
*Impatiens crenulata**	Shaanxi, China	Pei‐Liang Liu 1289 (WNU)	PX057469	PX067055	PX067124	PX067190	Q225
*Impatiens cyanantha*	Guizhou, China	SWFU‐IBLH20180817	—	MW464331			
*Impatiens cyanantha*	Yunnan, China	SWFU‐IBLH20180920	—	MW464332			
*Impatiens cyanantha**	Gansu, China	Pei‐Liang Liu 1743 (WNU)	PX057521	PX067107	PX067174	PX067242	Q322
*Impatiens cymbifera*	Xizang, China	S.X. Yu et al. 5629 (PE)	KP776069	KP776019	KP776128	—	
*Impatiens cymbifera*	Xizang, China	YS Chen et al. 706 (PE)	—	OR135515			
*Impatiens dasyvexilla**	Shaanxi, China	Pei‐Liang Liu 1734 (WNU)	PX057516	PX067102	PX067169	PX067237	Q316
*Impatiens davidii*	Fujian, China	H.N. Qin et al. 19960 (PE)	KP776070	—			
*Impatiens davidii*	Jiangxi, China	—	—	MZ424444			
*Impatiens delavayi*	Yunnan, China	Q Fan 19108 (PE)	—	OR135459			
*Impatiens delavayi*	Yunnan, China	Chassot & Yuan 99‐154 (NEU)	AY348773	—			
*Impatiens delavayi**	Yunnan, China	Pei‐Liang Liu 1429 (WNU)	PX057470	PX067056	PX067125	PX067191	Q228
*Impatiens devolii*	Taiwan, China	Jiang s. n.	AY348775	—			
*Impatiens dicentra*	Chongqing, China	B Ding BD201508032 (PE)	—	OR135441			
*Impatiens duclouxii*	Guangxi, China	S.X. Yu 4033 (PE)	KP776071	KP776021	KP776131	—	
*Impatiens ecornuta*	—	—	SRR19405685				
*Impatiens epilobioides*	Sichuan, China	WB Xu et al. Y12168 (PE)	—	OR135416			
*Impatiens faberi*	Sichuan, China	Song S007 (NEU)	AY348778	—			
*Impatiens faberi*	Sichuan, China	SX Yu 4087 (PE)	—	OR135485			
*Impatiens faberi**	Sichuan, China	Pei‐Liang Liu 1622 (WNU)	PX057491	PX067077	PX067144	PX067212	Q252
*Impatiens falcifer*	Xizang, China	S.X. Yu et al. 5659 (PE)	KP776072	KP776022	KP776133	—	
*Impatiens fanjingshanica*	Guizhou, China	SWFU‐IBFJS20171030	—	MW411294			
*Impatiens fargesii*	Chongqing, China	B Ding BD201509026 (PE)	—	OR135439			
*Impatiens fargesii**	Shaanxi, China	Pei‐Liang Liu 1736 (WNU)	PX057517	PX067103	PX067170	PX067238	Q317
*Impatiens fargesii**	Shaanxi, China	Pei‐Liang Liu 1738 (WNU)	PX057519	PX067105	PX067172	PX067240	Q319
*Impatiens fenghwaiana*	Jiangxi, China	Lushan Botanical Garden Expedition Team WNB2004‐00100 (PE)	—	OR135420			
*Impatiens fischeri*	Kenya, Africa	Odile Phaehler & Magali Schnell I‐01 (NEU)	AY348781	—			
*Impatiens fischeri*	Kenya, Africa	Menn. & Bar.‐Kui. 284 (U)	—	DQ147843	—	—	
*Impatiens fissicornis*	Shaanxi, China	Wang SH‐003 (NEU)	AY348782	—			
*Impatiens fissicornis*	Shaanxi, China	— I 0388 (PE)	—	OR135431			
*Impatiens fissicornis**	Shaanxi, China	Pei‐Liang Liu 429 (WNU)	PX057482	PX067068	PX067136	PX067203	Q243
*Impatiens forrestii*	Yunnan, China	Yunnan Expedition Team YN‐ET 1563 (PE)	—	OR135407			
*Impatiens forrestii*	Yunnan, China	Yuan CN2k‐79 (NEU)	AY348784	—			
*Impatiens fragicolor*	Xizang, China	S.X. Yu et al. 5318 (PE)	KP776073	KP776023	KP776134	—	
*Impatiens fujianensis*	Fujian, China	L. Ma 20231004 (FJFC)	PP889575	PP898334	PP898335	—	
*Impatiens furcillata*	Gangwon‐do, Korea	CH Nam, et al. N120191 (PE)	—	OR135429			
*Impatiens ganpiuana*	Guizhou, China	— 2107 (PE)	—	OR135507			
*Impatiens guiqingensis*	Gansu, China	CF Zhang 1275 (PE)	—	OR135514			
*Impatiens guiqingensis**	Shaanxi, China	Zai‐Min Jiang et al. 3652 (WUK)	PX057503	PX067090	—	—	Q276
*Impatiens harae*	Xizang, China	Y.S. Chen et al. 392 (PE)	KP776075	KP776025	KP776136	—	
*Impatiens henanensis*	Henan, China	Americans (s. n.) — (PE)	—	OR135445			
*Impatiens henryi*	Hubei, China	SX Yu et al. 8169 (PE)	—	OR135471			
*Impatiens huangyanensis*	Zhejiang, China	SX Yu et al. ZJ386 (PE)	—	OR135404			
*Impatiens hunanensis*	Guangxi, China	S.X. Yu 3759 (PE)	KP776077	KP776028	KP776137	—	
*Impatiens imbecilla*	Sichuan, China	Hao 426 (NEU)	AY348796	—			
*Impatiens kuntsunii* sp. nov.*	Sichuan, China	Pei‐Liang Liu 912 (WNU)	PX057455	PX067041	PX067110	PX067176	Q211
*Impatiens kuntsunii* sp. nov.*	Shaanxi, China	Pei‐Liang Liu 1321 (WNU)	PX057457	PX067043	PX067112	PX067178	Q213
*Impatiens kuntsunii* sp. nov.*	Shaanxi, China	Pei‐Liang Liu 424 (WNU)	PX057475	PX067061	PX067129	PX067196	Q233
*Impatiens kuntsunii* sp. nov.*	Gansu, China	Pei‐Liang Liu 869 (WNU)	PX057480	PX067066	PX067134	PX067201	Q239
*Impatiens kuntsunii* sp. nov.*	Shaanxi, China	Zhao‐Yang Chang et al. 2012184 (WUK)	PX057497	PX067083	PX067150	PX067218	Q268
*Impatiens kuntsunii* sp. nov.*	Shaanxi, China	Zai‐Min Jiang et al. 2640 (WUK)	—	PX067087	PX067154	PX067222	Q273
*Impatiens kuntsunii* sp. nov.*	Shaanxi, China	Zai‐Min Jiang et al. 2976 (WUK)	PX057501	PX067088	PX067155	PX067223	Q274
*Impatiens kuntsunii* sp. nov.*	Ningxia, China	Ren‐Bin Zhu 140 (WUK)	PX057505	—	PX067158	PX067226	Q278
*Impatiens kuntsunii* sp. nov.*	Gansu, China	Pei‐Liang Liu 1652 (WNU)	PX057511	PX067097	PX067164	PX067232	Q307
*Impatiens lacinulifera*	Gansu, China	Baishuijiang Expedition Team 1886 (PE)	—	OR135510			
*Impatiens lateristachys*	Sichuan, China	S.X. Yu et al. 4097 (PE)	KP776078	—			
*Impatiens lateristachys*	Sichuan, China	WB Xu et al. Y12231 (PE)	—	OR135414			
*Impatiens lateristachys**	Sichuan, China	Pei‐Liang Liu 1611 (WNU)	PX057488	PX067074	PX067141	PX067209	Q249
*Impatiens laxiflora*	Guangxi, China	S.X. Yu 3987 (PE)	KP776079	KP776031	KP776139	—	
*Impatiens lecomtei*	Yunnan, China	Yuan CN2k2‐202 (NEU)	AY348802	—			
*Impatiens lecomtei*	Xizang, China	XH Jin et al. DLJ‐ET 3303 (PE)	—	OR135432			
*Impatiens lecomtei**	Yunnan, China	Pei‐Liang Liu 1426 (WNU)	PX057486	PX067072	—	PX067207	Q247
*Impatiens leptocaulon*	Guangxi, China	S.X. Yu et al. 4019 (PE)	KP776080	KP776032	KP776140	—	
*Impatiens linghziensis**	Xizang, China	Pei‐Liang Liu 1567 (WNU)	PX057506	PX067092	PX067159	PX067227	Q279
*Impatiens linghziensis**	Xizang, China	Pei‐Liang Liu 1577 (WNU)	PX057507	PX067093	PX067160	PX067228	Q280
*Impatiens linocentra**	Shaanxi, China	Pei‐Liang Liu 1064 (WNU)	PX057466	PX067052	PX067121	PX067187	Q222
*Impatiens lixianensis*	Sichuan, China	SX Yu 3922 (PE)	—	OR135489			
*Impatiens lobulifera*	Guangxi, China	S.X. Yu et al. 3220 (PE)	KP776081	KP776033	KP776141	—	
*Impatiens longialata*	Chongqing, China	SX Yu et al. 8479 (PE)	—	OR135468			
*Impatiens longialata*	Chongqing, China	B Ding BD201509021 (PE)	—	OR135440			
*Impatiens lucorum*	Sichuan, China	WB Xu et al. Y12223 (PE)	—	OR135415			
*Impatiens lucorum**	Sichuan, China	Pei‐Liang Liu 1612 (WNU)	PX057489	PX067075	PX067142	PX067210	Q250
*Impatiens lucorum**	Sichuan, China	Pei‐Liang Liu 1619 (WNU)	PX057490	PX067076	PX067143	PX067211	Q251
*Impatiens macrovexilla*	Guangxi, China	S.X. Yu s.n. (PE)	KP776082	KP776034	KP776142	—	
*Impatiens macrovexilla* var. *yaoshanensis*	Guizhou, China	YB Luo 516 (PE)	—	OR135516			
*Impatiens malipoensis*	Guangxi, China	S.X. Yu 4037 (PE)	KP776083	KP776035	KP776143	—	
*Impatiens margaritifera*	Xizang, China	S.X. Yu et al. 6129 (PE)	KP776084	—			
*Impatiens menghuochengensis*	Sichuan, China	Q Luo 131015 (PE)	—	OR135449			
*Impatiens mengtszeana*	Yunnan, China	Yuan CN2k1‐60 (NEU)	AY348806	—			
*Impatiens mengtszeana*	Yunnan, China	SX Yu et al. 6314 (PE)	—	OR135479			
*Impatiens mexicana*	Oaxaca, Mexico	OA0029UAEH‐HGOM	OR525067	OR525131	OR525076	—	
*Impatiens microstachys*	Sichuan, China	S.X. Yu et al. 5043 (PE)	KP776085	—			
*Impatiens microstachys*	Sichuan, China	TT Xue Y12565 (PE)	—	OR135411			
*Impatiens monticola*	Yunnan, China	SWFU‐IBSD20180910	—	MW464334			
*Impatiens monticola*	Sichuan, China	Hao 425 (NEU)	AY348810	—			
*Impatiens morsei*	Guangxi, China	S.X. Yu 3127 (PE)	KP776086	KP776037	KP776145	—	
*Impatiens nasuta*	—	GanQL1461 (KUN)	MN862750	MN862751	MN862752	—	
*Impatiens nasuta*	Chongqing, China	B Ding BD201508012 (PE)	—	OR135443			
*Impatiens neglecta*	Anhui, China	H.N. Qin et al. 19942 (PE)	KP776087	KP776038	KP776147	—	
*Impatiens neglecta*	Anhui, China	HN Qin et al. 19942A (PE)	—	OR135457			
*Impatiens noli‐tangere*	Jilin, China	Yuan CN2k1‐86 (NEU)	AY348813	—			
*Impatiens noli‐tangere*	DNA barcoding the Flora of Qinling Mt. in China	—	MH710658	—			
*Impatiens noli‐tangere*	Zhejiang, China	HN Qin et al. 20090 (PE)	—	OR135455			
*Impatiens noli‐tangere**	Shanxi, China	Pei‐Liang Liu 1469 (WNU)	PX057453	PX067039	PX067108	PX067175	Q209
*Impatiens noli‐tangere**	Henan, China	Pei‐Liang Liu 1148 (WNU)	PX057467	PX067053	PX067122	PX067188	Q223
*Impatiens noli‐tangere**	Shanxi, China	Pei‐Liang Liu 1163 (WNU)	PX057478	PX067064	PX067132	PX067199	Q237
*Impatiens noli‐tangere**	Jilin, China	Ming‐Zhou Sun s.n. (WUK)	PX057481	PX067067	PX067135	PX067202	Q242
*Impatiens noli‐tangere**	Sinop, Turkey	Ali A. Dönmez 17663 (MO)	PX057496	PX067082	PX067149	PX067217	Q267
*Impatiens noli‐tangere**	Shaanxi, China	Zai‐Min Jiang et al. 3697 (WUK)	PX057504	PX067091	PX067157	PX067225	Q277
*Impatiens notolopha**	Sichuan, China	Pei‐Liang Liu 911 (WNU)	PX057454	PX067040	PX067109	—	Q210
*Impatiens notolopha**	Gansu, China	Zai‐Min Jiang et al. 1820 (WUK)	PX057500	PX067086	PX067153	PX067221	Q272
*Impatiens nubigena*	Sichuan, China	S.X. Yu et al. 4800 (PE)	KP776089	KP776040	KP776149	—	
*Impatiens nyimana*	Xizang, China	S.X. Yu et al. 6071 (PE)	KP776090	KP776041	KP776150	—	
*Impatiens obesa*	Guangxi, China	S.X. Yu 3775 (PE)	KP776091	KP776042	KP776151	—	
*Impatiens oxyanthera*	Sichuan, China	SX Yu 3269 (PE)	—	OR135500			
*Impatiens oxyanthera*	Sichuan, China	Song S008 (NEU)	AY348814	—			
*Impatiens pallida*	Ontario, Canada	—	MG217853	—			
*Impatiens pallida*	Ontario, Canada	—	MG219475	—			
*Impatiens pallida*	Tennessee, America	LR Phillippe et al. 42222 (PE)	—	OR135453			
*Impatiens paradoxa*	Henan, China	CS Zhu et al. 920312 (PE)	—	OR135448			
*Impatiens perforata* sp. nov.*	Gansu, China	Zai‐Min Jiang et al. 2024 (WUK)	PX057509	PX067095	PX067162	PX067230	Q292
*Impatiens perforata* sp. nov.*	Gansu, China	Zai‐Min Jiang et al. 3687 (WUK)	PX057510	PX067096	PX067163	PX067231	Q295
*Impatiens platyceras**	Gansu, China	Zai‐Min Jiang et al. 1767 (WUK)	PX057473	PX067059	—	PX067194	Q231
*Impatiens platychlaena*	Sichuan, China	SX Yu 3783 (PE)	—	OR135491			
*Impatiens platychlaena*	Sichuan, China	Song S009 (NEU)	AY348818	—			
*Impatiens platychlaena**	Sichuan, China	Pei‐Liang Liu 1610 (WNU)	PX057487	PX067073	PX067140	PX067208	Q248
*Impatiens platysepala*	Anhui, China	H.N. Qin et al. 19983A (PE)	KP776095	KP776044	KP776155	—	
*Impatiens plicatisepala*	Guangxi, China	SX Yu 4032 (PE)	—	OR135486			
*Impatiens poculifer*	Yunnan, China	Yuan CN2k2‐209 (NEU)	AY348820	—			
*Impatiens potaninii*	Chongqing, China	B Ding BD201508015 (PE)	—	OR135442			
*Impatiens potaninii*	DNA Barcoding the Flora of Qingling Mt. In China	—	MH808399	—			
*Impatiens potaninii**	Shaanxi, China	Pei‐Liang Liu 430 (WNU)	PX057465	PX067051	PX067120	PX067186	Q221
*Impatiens principis*	Guangxi, China	S.X. Yu 3749 (PE)	KP776096	KP776026	KP776156	—	
*Impatiens pterosepala*	Hubei, China	S.X. Yu s.n. (PE)	KP776097	KP776046	KP776158	—	
*Impatiens pterosepala*	Hubei, China	SX Yu et al. 8400 (PE)	—	OR135469			
*Impatiens pterosepala**	Shaanxi, China	Pei‐Liang Liu 1725 (WNU)	PX057512	PX067098	PX067165	PX067233	Q312
*Impatiens pterosepala**	Chongqing, China	Pei‐Liang Liu 1742 (WNU)	PX057520	PX067106	PX067173	PX067241	Q321
*Impatiens puberula*	Sagarmatha, Nepal	M Wakabayashi et al. 9715298 (PE)	—	OR135447			
*Impatiens pulchra*	Kachin state, Myanmar	Ruchisansakun et al. 751 (L)	MH377207	—			
*Impatiens purpurea*	Yunnan, China	Song Y007 (NEU)	AY348823	—			
*Impatiens quadriloba*	Sichuan, China	DE Boufford et al. 44474 (PE)	—	OR135452			
*Impatiens quadriloba**	Sichuan, China	Pei‐Liang Liu 907 (WNU)	PX057492	PX067078	PX067145	PX067213	Q255
*Impatiens racemosa*	Yunnan, China	S.X. Yu 4221 (PE)1 De Haas 2620 (U)2	KP776098	—			
*Impatiens radiata*	Xizang, China	De Haas 2620 (U)1 S.X. Yu et al. 4760 (PE)2	AY348824	—			
*Impatiens radiata*	Yunnan, China	XH Jin et al. ST2074 (PE)	—	OR135421			
*Impatiens rectirostrata*	Sichuan, China	SX Yu 3791 (PE)	—	OR135490			
*Impatiens reptans*	Hunan, China	SX Yu et al. M815 (PE)	—	OR135430			
*Impatiens rhombifolia*	Guangxi, China	SX Yu 3709 (PE)	—	OR135497			
*Impatiens rostellata**	Sichuan, China	Pei‐Liang Liu 1621 (WNU)	PX057479	PX067065	PX067133	PX067200	Q238
*Impatiens scabrida*	Xizang, China	S.X. Yu 5440 (PE)^1^	KP776099	—			
*Impatiens scullyi*	Xizang, China	Y.S. Chen et al. 652	KP776100	KP776048	KP776163	—	
*Impatiens serrata*	Xizang, China	L Wei et al. 15410 (PE)	—	OR135460			
*Impatiens shenglanii*	Hubei, China	Lixw 191001 (HIB)	MN685779	MN685777	MN685781	—	
*Impatiens shennongensis*	Hubei, China	SX Yu et al. 8149 (PE)	—	OR135472			
*Impatiens shimianensis*	Sichuan, China	Zhang & G. Zhang 4803 (CDBI MO)	HQ011920	—			
*Impatiens siculifer*	Yunnan, China	Yuan CN2k‐80 (NEU)	AY348831	—			
*Impatiens siculifer*	Guangxi, China	S.X. Yu 3211 (PE)	KP776101	—			
*Impatiens siculifera*	Yunnan, China	SX Yu et al. 6293 (PE)	—	OR135480			
*Impatiens soulieana*	Sichuan, China	SX Yu et al. WS‐346 (PE)	—	OR135419			
*Impatiens soulieana*	Sichuan, China	Yuan CN2k2‐163 (NEU)	AY348833	—			
*Impatiens* sp. Nanzheng*	Shaanxi, China	Pei‐Liang Liu 1205 (WNU)	PX057456	PX067042	PX067111	PX067177	Q212
*Impatiens* sp. Nanzheng*	Shaanxi, China	Pei‐Liang Liu 1304 (WNU)	PX057464	PX067050	PX067119	PX067185	Q220
*Impatiens* sp. Nanzheng*	Shaanxi, China	Pei‐Liang Liu 1252 (WNU)	PX057494	PX067080	PX067147	PX067215	Q259
*Impatiens stenosepala*	Shaanxi, China	Wang Sh001 (NEU)	AY348835	—			
*Impatiens stenosepala*	Guizhou, China	SWFU‐IBZE20171030	—	MW411298			
*Impatiens stenosepala**	Shaanxi, China	Pei‐Liang Liu 1267 (WNU)	PX057485	PX067071	PX067139	PX067206	Q246
*Impatiens subecalcarata*	Yunnan, China	Southeast Tibet Expedition Team SET_ET 70 (PE)	—	OR135424			
*Impatiens suichangensis*	Zhejiang, China	SX Yu et al. ZJ374 (PE)	—	OR135405			
*Impatiens sulcata*	Xizang, China	S.X. Yu et al. 5724 (PE)	KP776103	KP776051	KP776166	—	
*Impatiens sunkoshiensis*	Xizang, China	S.X. Yu et al. 5712 (PE)	KP776104	KP776052	KP776167	—	
*Impatiens sutchuenensis*	Chongqing, China	B Ding BD201508006 (PE)	—	OR135444			
*Impatiens sutchuenensis*	Chongqing, China	B Ding BD201509010 (PE)	—	OR135435			
*Impatiens sutchuenensis**	Shaanxi, China	Pei‐Liang Liu 1312 (WNU)	PX057474	PX067060	PX067128	PX067195	Q232
*Impatiens sutchuenensis**	Shaanxi, China	Pei‐Liang Liu 1233 (WNU)	PX057493	PX067079	PX067146	PX067214	Q257
*Impatiens sutchuenensis**	Hubei, China	Pei‐Liang Liu 1732 (WNU)	PX057514	PX067100	PX067167	PX067235	Q314
*Impatiens sutchuenensis**	Shaanxi, China	Pei‐Liang Liu 1733 (WNU)	PX057515	PX067101	PX067168	PX067236	Q315
*Impatiens tayemonii*	Taiwan, China	Z.Y. Jiang T2 (NEU)	AY348839	—			
*Impatiens tayemonii*	Taiwan, China	B Bartholomew et al. 14637 (PE)	—	OR135461			
*Impatiens textorii*	Japan	Kanno & al. 1114 (TUS)	AY348841	—			
*Impatiens textorii*	Gyeongsangnam, Korea	YH Cho et al. NAM‐08015‐058 (PE)	—	OR135428			
*Impatiens tienchuanensis*	Sichuan, China	SX Yu et al. WS‐761 (PE)	—	OR135417			
*Impatiens tortisepala*	Sichuan, China	S.X. Yu et al. 4925 (PE)	KP776106	KP776054	KP776170	—	
*Impatiens tortisepala**	Sichuan, China	Pei‐Liang Liu 1359 (WNU)	PX057495	PX067081	PX067148	PX067216	Q260
*Impatiens tuberculata*	Xizang, China	S.X. Yu et al. 5907 (PE)	KP776107	KP776055	KP776171	—	
*Impatiens uliginosa*	Yunnan, China	Yuan CN2k2‐173 (NEU)	AY348845	—			
*Impatiens uliginosa*	Yunnan, China	SWFU‐IBDSJF20180810	—	MN533984			
*Impatiens undulata*	Sichuan, China	Liden & Wang 2005‐3 (PE)	—	OR135509			
*Impatiens undulata**	Sichuan, China	Pei‐Liang Liu 1623 (WNU)	PX057460	PX067046	PX067115	PX067181	Q216
*Impatiens uniflora*	Taiwan, China	JR Chen 97504 (PE)	—	OR135450			
*Impatiens uniflora*	Taiwan, China	Zhengyu Jiang T1	AY348846	—			
*Impatiens urticifolia*	Xizang, China	S.X. Yu et al. 5630 (PE)1	KP776109	—			
*Impatiens vaughanii*	Satun, Thailand	Suksathan & Triboun 4843 (QBG)	KC905540	KC905575	—	—	
*Impatiens vittata*	Sichuan, China	WB Xu et al. Y12266 (PE)	—	OR135413			
*Impatiens wuyuanensis*	Jiangxi, China	XD Yang Y15156 (PE)	—	OR135408			
*Impatiens xanthocephala*	Sichuan, China	SX Yu et al. 9962 (PE)	—	OR135462			
*Impatiens yaoshanensis*	Sichuan, China	S.X. Yu et al. 4597 (PE)	KP776112	KP776059	KP776177	—	
*Impatiens yilingiana*	Zhejiang, China	X.F. Jin 1901 (HTC)	MN974566	MN974549	MN974583	—	
*Impatiens yunlingensis*	Sichuan, China	SX Yu et al. 9881 (PE)	—	OR135463			
*Impatiens zhui* sp. nov.*	Shaanxi, China	Pei‐Liang Liu 1631 (WNU)	PX057461	PX067047	PX067116	PX067182	Q217
*Impatiens zhuxiensis*	Hubei, china	Lixw 191662 (HIB)	MN823655	MN823654	MN823656	—	
*Impatiens zhuxiensis**	Hubei, China	Pei‐Liang Liu 1731 (WNU)	PX057513	PX067099	PX067166	PX067234	Q313

**Table:** Key to the new species and related species.

1. Flower solitary, axillary	2
1. Inflorescences often 2–5 flowered	4
2. Whole plant with indumentum	*I*. *dasyvexilla* Q. L. Gan & X. W. Li
2. Whole plant glabrous	3
3. Dorsal petal abaxial midvein carinate, near the top of the crest caudate	*I*. *zhui* Z. J. Huang & P. L. Liu
3. Dorsal petal abaxial midvein thickened, near the top of the crest not caudate	*I*. *pterosepala* Hook. f.
4. Lower sepal deeply saccate, base abruptly constricted into an incurved spur	5
4. Lower sepal infundibuliform or cornute, base gradually narrowed into a spur	7
5. Bract cupuliform, amplexicaul	*I. perforata* Z. J. Huang & P. L. Liu
5. Bract lanceolate	6
6. Corolla violet	*I*. *compta* Hook. f.
6. Corolla pink	*I. uncata* Y. Y. Cong & J. J. Zhou
7. Lamina ovate, oblong to suborbicular, apex often obtuse; lower sepal cornute	8
7. Lamina narrowly ovate to ovate, apex acute to acurminate; lower sepal infundibuliform	10
8. Corolla, lateral united petals and spur shorter than 1 cm	*I*. *aciformis* Z. J. Huang & P. L. Liu
8. Corolla, lateral united petals and spur longer than 1 cm	9
9. Dorsal petal abaxial midvein slightly carinate; spur curved but not into a circle	*I. notolopha* Maxim.
9. Dorsal petal abaxial midvein broadly carinate; spur winded into a circle	*I. undulata* Y. L. Chen & Y. Q. Lu
10. Lower sepal narrowly infundibuliform	*I*. *kunstunii* Z. J. Huang & P. L. Liu
10. Lower sepal broadly infundibuliform	11
11. Bract broadly ovate or orbicular	12
11. Bract linear, lanceolate or ovate	13
12. Bract margin dentate; lateral sepals asymmetrical	*I. latebracteata* Hook. f.
12. Bract margin entire; lateral sepals symmetrical	*I*. *cordibracteata* Z. J. Huang & P. L. Liu
13. Lamina base cuneate or attenuate; lateral sepals ovate	*I. noli‐tangere* L.
13. Lamina base rounded or cordate; lateral sepals orbicular or cordate	14
14. Bract linear or missing; upper lobe of lateral united petals dolabriform	*I*. *sutchuenensis* Franch. ex Hook. f.
14. Bract lanceolate to narrowly ovate; upper lobe of lateral united petals narrowly elliptic	*I. longialata* E. Pritz. ex Diels
